# Incidence, microbiological and immunological characteristics of ventilator-associated pneumonia assessed by bronchoalveolar lavage and endotracheal aspirate in a prospective cohort of COVID-19 patients: CoV-AP study

**DOI:** 10.1186/s13054-023-04658-5

**Published:** 2023-09-26

**Authors:** Davide Mangioni, Mauro Panigada, Emanuele Palomba, Chiara Bobbio, Liliane Chatenoud, Laura Alagna, Jacopo Fumagalli, Andrea Gori, Anna Grancini, Amedeo Guzzardella, Andrea Lombardi, Caterina Matinato, Andrea Meli, Antonio Muscatello, Laura Porretti, Mara Tomasello, Elena Trombetta, Luca Valenti, Alessandra Bandera, Giacomo Grasselli

**Affiliations:** 1 Infectious Diseases Unit, Foundation IRCCS Ca’ Granda Ospedale Maggiore Policlinico, Milan, Italy; 2 Department of Pathophysiology and Transplantation, University of Milano, Milan, Italy; 3 Department of Anaesthesia, Critical Care and Emergency, Foundation IRCCS Ca’ Granda Ospedale Maggiore Policlinico, Milan, Italy; 4 Istituto di Ricerche Farmacologiche Mario Negri IRCCS, Milan, Italy; 5 Microbiology Laboratory, Clinical Pathology, Foundation IRCCS Ca’ Granda Ospedale Maggiore Policlinico, Milan, Italy; 6 Flow Cytometry and Cell Sorting Laboratory, Clinical Pathology, Foundation IRCCS Ca’ Granda Ospedale Maggiore Policlinico, Milan, Italy; 7 Precision Medicine, Biological Resource Center Unit, Department of Transfusion Medicine, Foundation IRCCS Ca’ Granda Ospedale Maggiore Policlinico, Milan, Italy

**Keywords:** Intensive care unit, VAP, VALRTI, Molecular microbiology, Rapid diagnosis

## Abstract

**Background:**

No univocal recommendation exists for microbiological diagnosis of ventilator-associated pneumonia (VAP). Sampling of either proximal or distal respiratory tract likely impacts on the broad range of VAP incidence between cohorts. Immune biomarkers to rule-in/rule-out VAP diagnosis, although promising, have not yet been validated. COVID-19-induced ARDS made VAP recognition even more challenging, often leading to overdiagnosis and overtreatment. We evaluated the impact of different respiratory samples and laboratory techniques on VAP incidence and microbiological findings in COVID-19 patients.

**Methods:**

Prospective single-centre cohort study conducted among COVID-19 mechanically ventilated patients in Policlinico Hospital (Milan, Italy) from January 2021 to May 2022. Microbiological confirmation of suspected VAP (sVAP) was based on concomitant endotracheal aspirates (ETA) and bronchoalveolar lavage (BAL). Conventional and fast microbiology (FILMARRAY® Pneumonia Panel plus, BAL_FAPPP_) as well as immunological markers (immune cells and inflammatory cytokines) was analysed.

**Results:**

Seventy-nine patients were included. Exposure to antibiotics and steroid therapy before ICU admission occurred in 51/79 (64.6%) and 60/79 (65.9%) patients, respectively. Median duration of MV at VAP suspicion was 6 (5–9) days. Incidence rate of microbiologically confirmed VAP was 33.1 (95% CI 22.1–44.0) and 20.1 (95% CI 12.5–27.7) according to ETA and BAL, respectively. Concordance between ETA and BAL was observed in 35/49 (71.4%) cases, concordance between BAL_FAPPP_ and BAL in 39/49 (79.6%) cases. With BAL as reference standard, ETA showed 88.9% (95% CI 70.8–97.7) sensitivity and 50.0% (95% CI 28.2–71.8) specificity (Cohen’s Kappa 0.40, 95% CI 0.16–0.65). BAL_FAPPP_ showed 95.0% (95% CI 75.1–99.9) sensitivity and 69% (95% CI 49.2–84.7) specificity (Cohen’s Kappa 0.60, 95% CI 0.39–0.81). BAL IL-1β differed significantly between VAP (135 (IQR 11–450) pg/ml) and no-VAP (10 (IQR 2.9–105) pg/ml) patients (*P* = 0.03).

**Conclusions:**

In COVID-19 ICU patients, differences in microbial sampling at VAP suspicion could lead to high variability in VAP incidence and microbiological findings. Concordance between ETA and BAL was mainly limited by over 20% of ETA positive and BAL negative samples, while BAL_FAPPP_ showed high sensitivity but limited specificity when evaluating in-panel targets only. These factors should be considered when comparing results of cohorts with different sampling. BAL IL-1β showed potential in discriminating microbiologically confirmed VAP.

*Clinical Trial registration*: NCT04766983, registered on February 23, 2021.

**Supplementary Information:**

The online version contains supplementary material available at 10.1186/s13054-023-04658-5.

## Introduction

Ventilator-associated pneumonia (VAP) is the most frequent infection in the intensive care unit (ICU) and one of the major complications associated with invasive mechanical ventilation (MV). Patients with VAP face longer MV, prolonged ICU stay and possibly poorer outcomes [1]. Incidence of VAP ranges broadly from 5% up to 40% [1], partly due to the use of multiple diagnostic algorithms without a univocal gold standard [2–5].

Depending on guidelines, microbiological diagnosis is recommended either by proximal respiratory samples (endotracheal aspirate, ETA) or by distal sampling (bronchoalveolar lavage, BAL) [6, 7], which sampling method to use is still debated. While ETA is less expensive and easier to perform under challenging situations such as severe acute respiratory distress syndrome (ARDS), BAL provides a larger sample for adjunctive analyses (*i.e.,* viruses or fungi detection, immunological analyses) and is characterized by higher specificity for microbiological confirmation of suspected VAP [1, 3]. This is a crucial issue since VAP has been recognized as a significant driver of antibiotic use in ICU [8]. Misdiagnosis of VAP can lead to antibiotics misuse, either as overtreatment of suspected cases or as employment of broad-spectrum antibiotics, all with significant ecological impact in terms of multidrug-resistant organisms (MDROs) selection [1, 3].

From an immunological standpoint, high levels of pro-inflammatory cytokines and immune cell markers have been observed both in BAL and peripheral blood (PB) of patients with microbiologically confirmed VAP [4, 9, 10]. Some of these markers have been proposed as adjunctive tools in suspected VAP to avoid antibiotic exposure for unconfirmed cases, with conflicting results [11].

Recently, coronavirus disease 2019 (COVID-19)-induced ARDS has made VAP recognition and management even more challenging. Factors associated with SARS-CoV-2 infection itself and its treatment contributed, on the one hand, to higher incidence of VAP. On the other, they directly impacted major clinical, laboratory and radiologic parameters employed in VAP diagnosis [12]. VAP has been reported in up to 79% of mechanically ventilated COVID-19 patients [13], with a high incidence of MDROs [14]. Accurate recognition of microbiologically confirmed VAP is of utmost importance in this setting.

Goals of the present study were: (i) to assess incidence of VAP by ETA and BAL performed simultaneously at VAP suspicion in COVID-19 ICU patients; (ii) to compare microbial isolates and evaluate concordance between sampling methods and diagnostic techniques (conventional culture vs molecular microbiology); and (iii) to examine immune cell and cytokines in BAL and PB of patients with VAP.

## Methods

### Study design and population

Single-centre cohort study conducted in the ICU of Policlinico Hospital (Milan, Italy). The study included two phases, a retrospective and a prospective cohort.

All consecutive patients requiring MV for ARDS in laboratory-confirmed SARS-CoV-2 infection were considered. Exclusion criteria were age < 18 years, total length of MV ≤ 48 h or MV ongoing for > 48 h at enrolment, and lack of comprehensive clinical documentation.

The study was registered by the Milan Area 2 Ethical Committee (#97_2021) and was conducted following standards of the Helsinki Declaration. The study was registered at clinicaltrials.gov on 23 February 2021 (NCT04766983).

The present analysis is focused on the prospective cohort only (details of the overall study design and aims are accessible on clinicaltrials.gov). In the prospective cohort, all consecutive COVID-19 ICU patients admitted from 21 January 2021 to 17 May 2022 were included. As soon as VAP was clinically suspected, patients underwent collection of ETA immediately followed by collection of BAL, following institutional guidance (see Additional file 1).

### Laboratory analyses and data collection

Microbial samples were analysed for quantitative cultures on ETA and BAL, for rapid molecular microbiology (BIOFIRE® FILMARRAY® Pneumonia Panel plus, BAL_FAPPP_) on BAL only. Immunological samples were analysed for immune cells and cytokines (Human Magnetic Luminex® custom assay) on BAL and PB (see Additional file 1).

Demographic, clinical, laboratory and outcome data were collected from clinical records using REDCap (Research Electronic Data Capture).

### VAP definition

VAP was suspected (sVAP) *as per* clinical practice in the presence of new or progressive radiographic infiltrates (if available) plus at least two between fever > 38 °C, leucocytosis (leucocytes > 10.8 μL) or leukopenia (leucocytes < 4.8/µL), purulent tracheobronchial secretions and respiratory deterioration without any discernible cause [3, 7]. Each sVAP event was reviewed by dedicated intensivists and infectious disease (ID) specialists (DM, JF, AMe). Discordances were subjected to collegial evaluation with senior physicians of the CoV-AP study group (MP, AB).

Microbiological confirmation of sVAP was based on the results of diagnostic ETA/BAL at cut-offs of ≥ 10^5^ CFU/ml for ETA and ≥ 10^4^ CFU/ml for BAL (see Additional file 1) [3, 7]. Non-pathogenic organisms such as coagulase-negative staphylococci, enterococci and *Candida* spp. were deemed insignificant, irrespectively from quantitative culture results. *Aspergillus* spp. isolation was always considered significant given the high-risk setting for COVID-19-associated pulmonary aspergillosis (CAPA) [15]. The microbiological cut-off for BAL_FAPPP_ was set at 10^4^ copies/mL in accordance with FDA clearance [16]. MDROs were defined as resistant to at least one agent in three or more antimicrobial categories or when harbouring specific antibiotic resistance mechanisms (e.g., methicillin-resistant *Staphylococcus* spp, ESBL/carbapenemases-producing *Enterobacterales*) using rapid detection methods [17, 18]. Secondary bloodstream infections (BSI) were defined using the secondary BSI attribution period according to the Centers for Diseases Control and Prevention National Healthcare Safety Network (CDC-NHSN) [19]. In case of discrepancies between ETA and BAL results, clinical decisions were taken on case-by-case basis after confrontation between ICU physician and ID consultant. Immunological analyses on BAL and PB were not considered for the clinical diagnosis of sVAP nor for VAP confirmation, in accordance with the most recent definitions of VAP [2–7].

### Statistical analysis

Patients' demographic, clinical, and laboratory characteristics, along with other variables of interest, were described using median and quartiles (Q1-Q3) or frequencies and proportions, depending on their distribution.

Incidence rate (IR_VAP_ per 1000 patient-MVdays) of the first VAP event was calculated from MV start to VAP diagnosis, MV end, or death, whichever came first. Sensitivity, specificity, positive and negative predictive values of ETA and BAL_FAPPP_ were calculated considering BAL results as reference standard. Cohen's kappa coefficient was used to assess agreement between tests (ETA vs BAL, BAL_FAPPP_ vs BAL). Details in Additional file 1.

## Results

### Population description

**Fig. 1:**
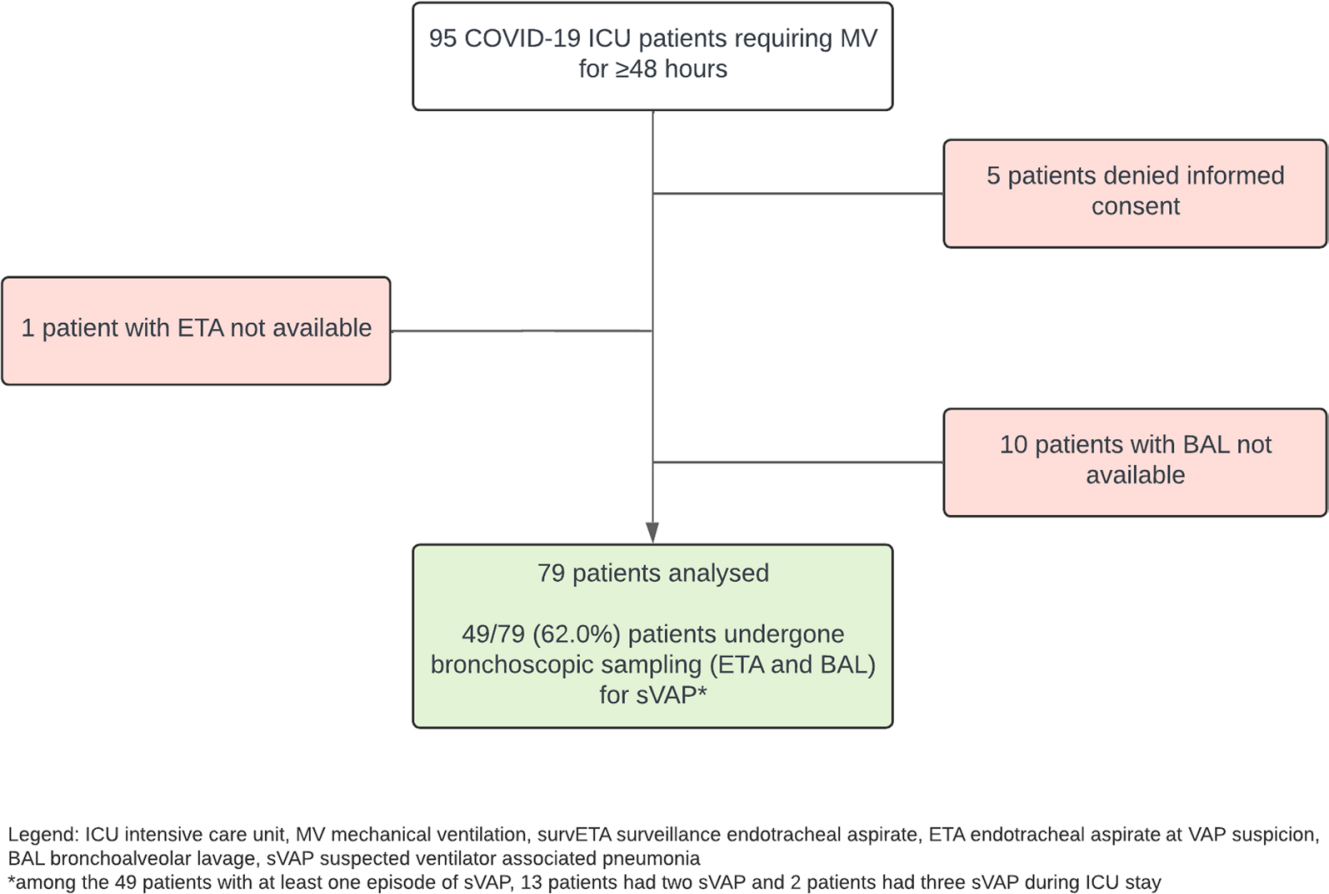
Study flow chart

Of the 95 patients enrolled, 5 were excluded due to consent withdrawal, and 11 further excluded for principal analyses due to incomplete collection of ETA or BAL at sVAP. Of the retained 79 patients, 49 (62%) had at least 1 episode of sVAP with concomitant ETA and BAL collection. Study flow chart is represented in Fig. 1, patients’ enrolment by month in Additional file 1: Fig. S1.

Clinical characteristics and outcomes of the study cohort are reported in Table 1, overall and for sVAP and no-sVAP patients. Groups did not differ for demographic features nor severity characteristics at ICU admission Exposure to antibiotics and steroid therapy before admittance occurred in most patients (51/79 (64.6%) and 60/79 (65.9%), respectively) with no differences between groups. Only 9/79 (11.4%) patients had a documented bacterial infection before ICU admission, with antibiotic therapy stopped at admission in all but two cases. Median duration of MV at VAP suspicion was 6 (5–9) days. Among the 49 patients with sVAP, 9 (18.4%) and 20 (40.8%) had ongoing antibiotics and steroid therapy at VAP suspicion, respectively.

**Table 1:** Clinical characteristics and outcomes of the 79 patients enrolled with both ETA and BAL collected at VAP suspicion

	Total N = 79	Suspected VAP N = 49	no-VAP suspicion N = 30	*P* value
Demographic characteristics				
Gender male	55 (69.6)	35 (71.4)	20 (66.7)	0.66
Age, years	60.0 (52.0–67.0)	61.0 (53.0–66.0)	58.5 (49.0–67.0)	0.46
BMI	27.8 (24.8–32.7)	27.7 (25.2–32.7)	27.8 (24.6–30.1)	0.73
CCI				
0	54 (68.4)	33 (67.4)	21 (70.0)	0.85
1	17 (21.5)	11 (22.4)	6 (20.0)	
2–3	8 (10.1)	5 (10.2)	3 (10.0)	
Clinical characteristics pre-ICU admission				
Days from symptoms onset to hospitalization	6.0 (4.0–8.0)	5.0 (3.0–7.0)	6.0 (5.0–9.0)	0.16
Documented bacterial infections	9 (11.4)	7 (14.3)	2 (6.7)	0.47
CAP/HAP	3 (3.8)	3 (6.1)	0 (0.0)	
Primary BSI/CRBSI	2 (2.5)	2 (4.1)	0 (0.0)	
UTI	4 (5.1)	2 (4.1)	2 (6.7)	
Exposure to antibiotic therapy	51 (64.6)	28 (57.1)	23 (76.7)	0.08
Exposure to steroid therapy	60 (75.9)	37 (75.5)	23 (76.7)	0.91
Standard^a^	39 (49.4)	23 (46.9)	16 (53.3)	
High dose^b^	12 (15.2)	7 (14.3)	5 (16.7)	
Both	7 (8.9)	5 (10.2)	2 (6.7)	
Exposure to other immunosuppressive therapy before admission^c^	12 (15.2)	6 (12.2)	6 (20.0)	0.35
Clinical characteristics at ICU admission				
Days from hospitalization to MV start	4.0 (1.0–7.0)	4.0 (3.0–9.0)	3.0 (1.0–5.0)	0.04
SOFA score	6.0 (4.0–7.0)	6.0 (4.0–8.0)	6.0 (4.0–7.0)	0.85
PaO_2_: FiO_2_ ratio	101.0 (73.0–120.0)	102.0 (73.0–124.0)	100.5 (74.0–120.0)	0.97
Leucocytes count, 10^3^ cell/*µ*L	9.6 (7.6–13.4)	9.9 (7.9–13.3)	9.0 (7.1–14.7)	0.53
C reactive protein, mg/dl	7.8 (3.9–17.7)	7.0 (3.9–17.5)	9.8 (4.6–19.9)	0.30
Procalcitonin, *µ*g/L	0.2 (0.1–0.5)	0.2 (0.1–0.5)	0.2 (0.1–0.7)	0.73
Clinical characteristics at VAP suspicion				
Days from MV start to VAP suspicion	–	6.0 (5.0–9.0)	–	–
Ongoing antibiotic therapy	–	9 (18.4)	–	–
Ongoing steroid therapy	–	20 (40.8)	–	–
Standard^a^		17 (34.7)		
High dose^b^		3 (6.1)		
Prone position	–	23 (46.9)	–	–
ECMO support	–	3 (6.1)	–	–
SOFA score	–	5.0 (3.0–6.0)	–	–
PaO_2_: FiO_2_ ratio	–	136 (109–162)	–	–
Leucocytes count, 10^3^ cell/*µ*L	–	11.0 (8.5–14.3)	–	–
C reactive protein, mg/dl	–	16.0 (10.9–20.0)	–	–
Procalcitonin, µg/L	–	0.3 (0.2–0.6)	–	–
MR-proADM, nmol/L	–	1.2 (0.9–1.6)	–	–
Secondary BSI	–	9 (18.4)	–	–
Outcome characteristics				
MV duration, days	18.0 (9.0–30.0.)	24.0 (16.0–44.0)	7.5 (5.0–13.0)	< .01
ICU length of stay, days	19.0 (10.0–33.0)	27.0 (17.0–55.0)	9.0 (6.0–15.0)	< .01
ICU mortality	26 (32.9)	20 (40.8)	6 (20.0)	0.056

*BMI* body mass index, *CCI* Charlson comorbidity index (age-unadjusted), *ICU* intensive care unit, *CAP* community-acquired pneumonia, *HAP* hospital-associated pneumonia, *BSI* bloodstream infection, *CRBSI* catheter-related bloodstream infection, *UTI* urinary tract infection, *MV* mechanical ventilation, *VAP* ventilator associated pneumonia, *ECMO* extracorporeal membrane oxygenation, *SOFA* score sequential [sepsis-related] organ failure assessment score

^a^Standard: dexamethasone (any dosage) or methylprednisolone ≤ 1 mg/kg/day

^b^High dose: methylprednisolone > 1 mg/kg/day

^c^among patients with no-VAP suspicion, 3 (10.0%) assumed Baricitinib and 3 (10.0%) assumed Tocilizumab; among patients with VAP suspicion, 4 (8.2%) assumed Baricitinib and 2 (4.1%) assumed Tocilizumab

Outcome measures differed significantly between groups, with median duration of MV of 24 (16–44) days in sVAP and 7.5 (5–13) days in no-sVAP (*P* < 0.001), ICU length of stay of 27 (17–55) days in sVAP and 9 (6–15) days in no-sVAP (*P* < 0.001). Overall ICU mortality was 26/79 (32.9%), with relevant differences between groups albeit not reaching statistical significance (20/49 (40.8%) in sVAP and 6/30 (20%) in no-sVAP, *P* = 0.056).

Analysis including patients undergone incomplete bronchoscopy sampling (*i.e.,* ETA or BAL) is reported in Additional file 1: Table S1. Comparisons within the sVAP group by results of ETA and BAL are reported in Additional file 1: Tables S2-S4.

### VAP incidence by BAL and ETA

Incidence of microbiologically confirmed VAP varied significantly depending on microbial sampling (Table 2).

**Table 2:** Incidence rate of microbiologically confirmed VAP according to different respiratory sampling techniques

BAL	
Positive test, n (%)	27/79 (34%)
Time at risk (days of MV)	1343
IR_VAP_ (95% CI)	20.1 (12.5–27.7)

ETA	
Positive test, n (%)	35/79 (44%)
Time at risk (days of MV)	1058
IR_VAP_ (95% CI)	33.1 (22.1–44.0)

*MV* mechanical ventilation, IR_VAP_ incidence rate of microbiologically confirmed VAP per 1000 ventilator days

The proportion of VAP by BAL and ETA was 27/79 (34%) and 35/79 (44%), respectively. IR_VAP_ according to BAL was 20.1 (95% CI 12.5–27.7) per 1000 ventilator days; according to ETA, it increased to 33.1 (95% CI 22.1–44.0) per 1000 ventilator days.

### Microbiological diagnosis of VAP and comparison between samples

**Fig. 2:**
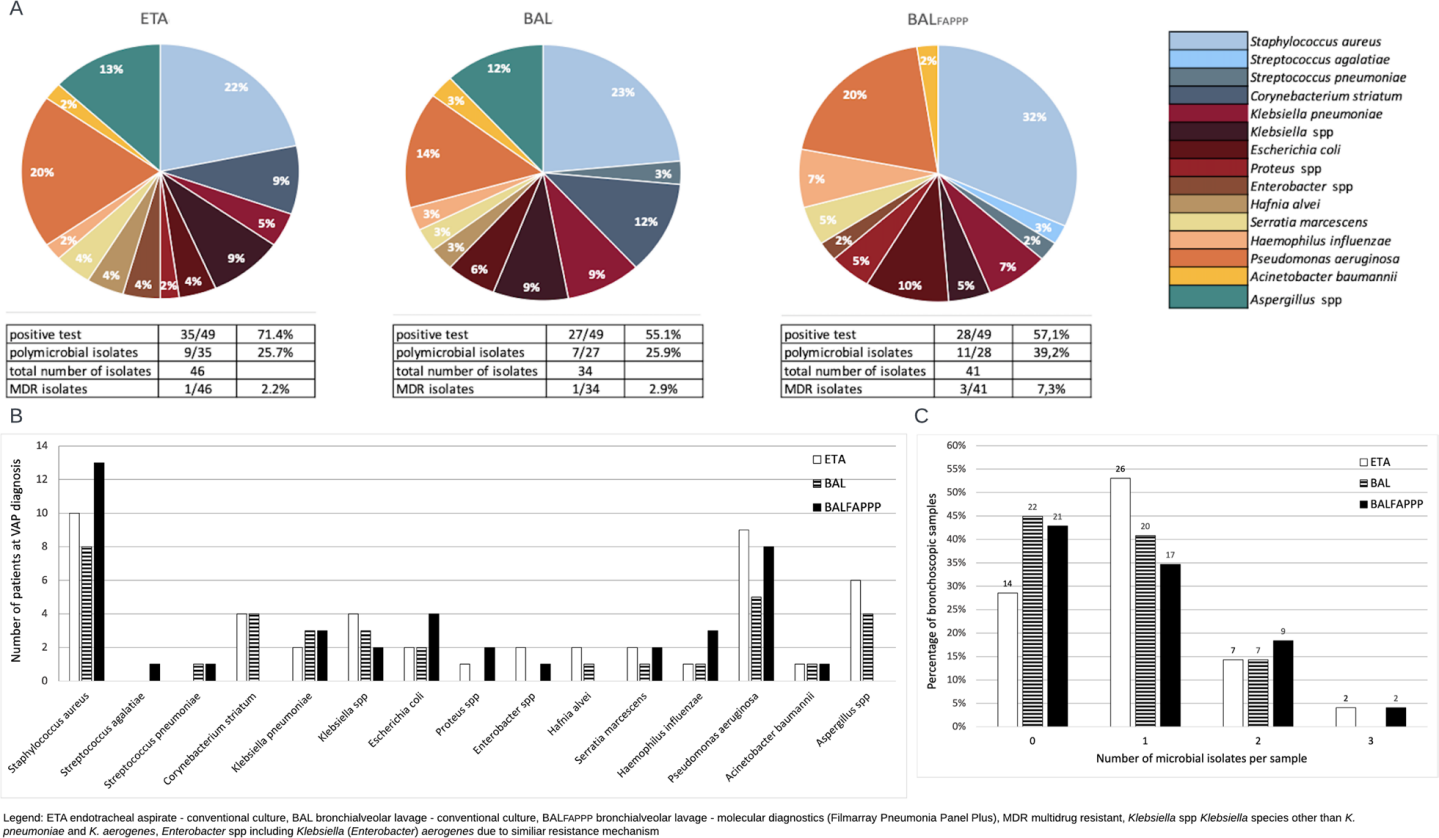
Microbial isolates of VAP episodes confirmed by ETA, BAL and BAL_FAPPP_. Panels A and B: proportion (**A**) and frequency (**B**) of isolated pathogens among different bronchoscopic samples. Panel **C**: number of microbial isolates per sample

Microbial isolates according to different respiratory samples and diagnostic techniques are reported in Fig. 2 and Additional file 1: Table S5-S6. Polymicrobial isolates were found in a quarter of positive samples assessed by conventional culture (25.7% (9/35) ETA, 25.9% (7/27) BAL), and increased to 39.2% (11/28) with BAL_FAPPP_. *Staphylococcus aureus* (21.7% (10/46) ETA, 23.5% (8/34) BAL, 31.7% (13/4) BAL_FAPPP_) and *Pseudomonas aeruginosa* (19.6% (9/46) ETA, 14.7% (5/34) BAL, 19.5% (8/41) BAL_FAPPP_) were the most frequent isolates. Notably, a substantial proportion of conventional cultures resulted in positive for *Aspergillus* spp. (13.0% (6/46) ETA, 11.8% (4/34) BAL) and *Corynebacterium striatum* (8.7% (4/46) ETA, 11.8% (4/34) BAL), which are not detectable by BAL_FAPPP_.

Prevalence of MDROs was low (2.2% (1/46) in ETA, 2.9% (1/34) in BAL and 7.3% (3/41) in BAL_FAPPP_).

Overall, secondary BSI was documented in 9 cases, specifically in 8/35 (22.8%) VAP according to ETA and 7/27 (25.9%) VAP by BAL. Details are reported in Additional file 1: Table S7.

### Concordance between diagnostic techniques

Concordance between test results and microbial isolates are reported in Table 3, details in Additional file 1: Table S8-S9.

**Table 3:** Agreement analysis between different respiratory samples (ETA *vs* BAL) and diagnostic techniques (BAL_FAPPP_ *vs* BAL) of suspected VAP

	ETA	BAL	FN	FP	TN	TP	% sensitivity (95% CI)	% specificity (95% CI)	Cohen’s Kappa (95% CI)
ETA vs BAL concordance									
Positive test (any species)	35	27	3	11	11	24	88.9 (70.8–97.7)	50.0 (28.2–71.8)	0.40 (0.16–0.65)
*Staphylococcus aureus*	10	8	1	3	38	7	87.5 (47.3–99.7)	92.7 (80.1–98.5)	0.73 (0.48–0.98)
*Pseudomonas aeruginosa*	9	5	0	4	40	5	100.0 (47.8–100.0)	90.9 (78.3–97.5)	0.67 (0.38–0.96)

	BAL_FAPPP_	BAL	FN	FP	TN	TP	% sensitivity (95% CI)	% specificity (95% CI)	Cohen’s Kappa (95% CI)
BAL_FAPPP_ vs BAL concordance*									
Positive test (any species)	28	20	1	9	20	19	95.0 (75.1–99.9)	69.0 (49.2–84.7)	0.60 (0.39–0.81)
*Staphylococcus aureus*	13	8	0	5	36	8	100.0 (63.1–100.0)	87.8 (73.8–95.9)	0.70 (0.47–0.94)
*Pseudomonas aeruginosa*	8	5	0	3	41	5	100.0 (47.8–100.0)	93.2 (81.3–98.6)	0.74 (0.46–1.00)

Conventional culture of BAL is considered the reference standard. Categorical concordance based on test results and microbial concordance of the two most frequently isolated pathogens (*Staphylococcus aureus* and *Pseudomonas aeruginosa*) are reported

*ETA* endotracheal aspirate-conventional culture, *BAL* bronchoalveolar lavage-conventional culture, *BAL*_FAPPP_ bronchoalveolar lavage-molecular diagnostics (FILMARRAY® Pneumonia Panel plus), *FN* false negative, *FP* false positive, *TN* true negative, *TP* true positive

*For concordance purpose, the 7 microbiologically confirmed VAP with detection in BAL of isolates not identifiable by BAL_FAPPP_ were considered BAL negative

In 35/49 (71.4%) we observed concordance between results of paired ETA and BAL, with both samples positive in 24/49 (49%) and negative in 11/49 (22.4%). Discordance was mainly due to ETA positivity and BAL negativity (11/49, 22.4%), while the opposite was found in 3/49 (6.1%) cases. With BAL as reference standard, ETA had 88.9% sensitivity (95% CI 70.8–97.7), 50.0% specificity (95% CI 28.2–71.8), leading to a fair agreement (Cohen’s Kappa 0.40, 95% CI 0.16–0.65).

For concordance purpose between BAL_FAPPP_ and BAL, the 7 microbiologically confirmed VAP with detection in BAL culture of isolates not identifiable by BAL_FAPPP_ were considered BAL negative. Concordance between BAL_FAPPP_ and BAL was obtained in 39/49 (79.6%), with both samples positive in 19/49 (38.8%), negative in 20/49 (40.8%). Discordance was mainly due to BAL_FAPPP_ positivity and BAL negativity (9/49, 18.4%), while the opposite was found in 1/49 (2.0%) case. BAL_FAPPP_ performed with 95.0% sensitivity (95% CI 75.1–99.9), 69% specificity (95% CI 49.2–84.7), leading to moderate agreement (Cohen’s Kappa 0.60, 95% CI 0.39–0.81).

We then evaluated microbial concordance between tests for *Staphylococcus aureus* and *Pseudomonas aeruginosa*. Concordances of both ETA vs BAL and BAL_FAPPP_ vs BAL resulted higher, with Cohen’s Kappa for *Staphylococcus aureus* of 0.73 (95% CI 0.48–0.98) and 0.70 (95% CI 0.47–0.94), for *Pseudomonas aeruginosa* of 0.67 (95% CI 0.38–0.96) and 0.74 (95% CI 0.46–1.00), respectively (Table 3).

### Immune markers of suspected VAP in BAL and PB

Major findings of immune cells and cytokines in BAL and PB of the 49 sVAP patients with and without VAP according to BAL results are reported in Additional file 1: Fig. S2, details in Additional file 1: Tables S10-S11.

In BAL, VAP patients were characterized by higher proportion of neutrophils (85.2% *vs* 66.7%, crude *P* = 0.01) and lower proportion of lymphocytes-monocytes (lymphocytes 2% *vs* 7.2%, crude *P* = 0.03; monocytes 5.4% *vs* 9%, crude *P* = 0.03) compared to no-VAP patients (Additional file 1: Fig. S2, Panel A).

BAL IL-1β differed significantly between VAP (135 (11–450) pg/ml) and no-VAP (10 (2.9–105) pg/ml) patients, crude *P* = 0.03 (Additional file 1: Fig. S2, Panel B). At its best cut-off value of 7.9 pg/ml, IL-1β performance for VAP diagnosis resulted in 81.5% sensitivity (95% CI 61.9–93.7), 50.0% specificity (95% CI 27.2–72.8), 68.8% positive predictive value (95% CI 50.0–83.9), and 66.7% negative predictive value (95% CI 38.4–88.2).

In both BAL and PB, CXCL-10 and IL-6 levels did not differ significantly between groups (Additional file 1: Table S11).

## Discussion

We demonstrated in a prospective cohort of COVID-19 ICU patients different incidences of VAP and heterogeneity in microbial findings depending on the respiratory sampling (ETA *vs* BAL) and the diagnostic technique employed (molecular microbiology *vs* conventional culture). Moreover, we proposed BAL IL-1β as a promising tool for supporting VAP diagnosis in COVID-19 patients.

The negative impact of VAP on COVID-19 patients’ outcomes is well established. While VAP influence on the length of MV and ICU stay has been documented, its impact on mortality is still debated [20, 21]. Le Pape et al. recently published a retrospective cohort study on ARDS due to COVID-19 or other conditions [20]. In COVID-19 patients, VAP was associated with longer ICU stay but did not predict death. Different results were obtained by Nseir et al., who reported a 1.7-fold increase in VAP-related mortality in COVID-19 patients but not in patients without SARS-CoV-2 infection [21]. Similarly, in their multicentre study across 149 European COVID-19 ICUs, Garnier et al. found an association of VAP with 90-day mortality with a hazard ratio of 1.34 [22].

Consistently with these data, in our prospective cohort VAP significantly impacted patients’ outcomes. Despite our study was not designed for this purpose and was possibly underpowered in sample size population, lengths of MV and ICU stay were three times longer in patients with suspected VAP than in those with no VAP suspicion (median of 24 *vs* 7.5 days and 27 *vs* 9 days, respectively) and ICU mortality doubled (40.8% vs 20%). Interestingly, we found a twofold increase in risk of death in case of positive bronchoscopy sample in the subgroup of patients with suspected VAP who underwent respiratory sampling (45.7% *vs* 28.6% ICU mortality according to ETA and 51.8% *vs* 27.3% according to BAL results). Although not statistically significant, these findings may suggest that accurate microbial confirmation of suspected VAP helps to distinguish actual infectious episodes, which have the greatest impact on patient outcomes.

VAP incidence varies considerably between studies. In ICU patients without COVID-19, it has been reported in 5% to 40% of the population, with incidence rate between 2.5 and 18.3 per 1000 ventilation days [1, 23, 24]. The risk increases in COVID-19 patients, with VAP reported in 18% to 79% [13, 25] and incidence rate ranging between 13.5 and 48.8 per 1000 ventilation days [26, 27]. Of note, VAP incidence is influenced by the criteria used for its definition and varies widely even when analysing the same population [2]. A recent systematic review on VAP diagnostics reported that microbiological analysis was required in almost three-quarters of studies [4]. However, which sampling method to use is still controversial [6, 7] and scarce evidence is available on variation in VAP incidence according to the type of microbial sample.

In our cohort, incidence rate of microbiologically confirmed VAP was 33.1 per 1000 ventilator days by ETA and 20.1 per 1000 ventilator days by BAL, with a proportion of 44% and 34% cases, respectively. Likewise, concordance between test results was observed in less than three-quarters of cases. Considering BAL as reference test, ETA showed good sensitivity but limited specificity, in line with previous studies in patients without COVID-19 [3, 4].

VAP aetiology in our cohort was in line with studies conducted by our group and others during the first COVID-19 pandemic wave [18, 26, 28]. However, we found MDROs in less than 3% of VAP with conventional culture and 7.3% with molecular microbiology, much lower than what was reported in previous studies by Grasselli et al. in Italy and Moreno et al. in France at 35% and 28% respectively [18, 28]. Implementing antimicrobial stewardship (AMS) and preventive strategies both prior to and during the ICU stay has likely contributed to sharply reduce the burden of MDROs across different pandemic waves [14, 29].

Our results allow us to directly compare rapid molecular microbiology to conventional cultures for VAP diagnosis in COVID-19 BAL samples. Multiplex PCR-based testing is emerging as a tool that could potentially transform the management of suspected VAP. It offers high sensitivity and short turnaround time, showing great potential for optimizing therapy and improving antibiotic stewardship [30, 31]. BAL_FAPPP_ has already proved to be highly concordant to conventional culture (> 90%) in diagnosing bacterial coinfection among COVID-19 patients at ICU admittance [32]. In our cohort, its primary clinical limitation was related to the relevant proportion of *Aspergillus* spp. and to the outbreak of VAP by *Corynebacterium striatum* that occurred during the study period, which BAL_FAPPP_ cannot detect. These could be possibly related to the high proportion of patients exposed to steroid therapy before ICU admission and at the time of VAP suspicion (over 75% and 40%, respectively). When evaluating in-panel targets only, BAL_FAPPP_ showed high sensitivity but limited specificity considering BAL conventional cultures as reference standard. This is in line with results of the INHALE study conducted in patients without SARS-CoV-2 infection, which found BAL_FAPPP_ sensitivity of 91.7–100.0% and specificity of 87.5–99.5%, depending on bacterial species [30]. We can suggest BAL_FAPPP_ as a helpful tool to rule out bacterial VAP in ICU patients with severe viral infections such as COVID-19 and avoid inappropriate antibiotic therapies. Positive tests should yet be interpreted wisely, based on patients’ clinical signs, previous microbial findings, and local ecology.

Besides molecular microbiology, the immune response of VAP patients is being studied with great interest to sought for rapid and accurate diagnostic markers [4]. Among non-COVID-19 patients, IL-1β has been proposed as promising markers for VAP [9, 10]. Conway-Morris et al. have demonstrated at the cut-off of 10 pg/ml an area under the curve (AUC) of 0.81 (95% CI 0.71–0.91) to predict VAP, with 94% sensitivity and 64% specificity [9]. Similar performance was shown by Hellyer et al., who found at the cut-off of 17 pg/ml an AUC of 0.81 (95% CI 0.74–0.88). A combination of IL-1β and IL-8 at the optimal cut-points excluded VAP with a sensitivity of 100% and specificity of 44.3% and was proposed as a rapid biomarker-based rule-out test with the potential to improve antibiotic stewardship [10]. Consistently with these data, in our prospective cohort, BAL IL-1β showed potential in discriminating microbiologically confirmed VAP within a larger population of patients with VAP suspicion, with 81.5% sensitivity and 50% specificity at the cut-off point of 7.9 pg/ml.

However, the possible impact of rapid host response markers on AMS remains to be proved. The VAPrapid2 trial conducted among non-COVID-19 patients at VAP suspicion found no impact on antibiotic consumption of therapy guided by BAL IL-1β and IL-8 measurement compared to routine management [11]. The authors suggested that the antibiotic prescribing behaviour and the lack of technology adoption significantly influenced trial outcomes. As already proved in BSI, novel rapid diagnostics are likely to impact on clinical outcomes only when integrated in a structured AMS framework [33].

This study has limitations. Firstly, its monocentric design may hamper the generalizability of the results. Nevertheless, characteristics of our cohort were consistent with COVID-19 ICU patients described in previous studies, and respiratory samples were taken according to well-standardized and reproducible methods. Therefore, we believe that our population well represents mechanically ventilated patients with severe COVID-19, while caution should be taken when translating our results to patients without SARS-CoV-2. Secondly, subgroup of patients with ETA and BAL collected simultaneously at VAP suspicion was limited and did not allow to draw conclusions on the impact of VAP on clinical outcome. Similarly, immunological analyses and microbial concordance may have required a larger sample size, considering the relevant proportion of patients on immunosuppressive and antibiotic therapies. These were not primary goals of our study, which reflects the real-life scenario with difficulty in obtaining distal sampling in patients with severe ARDS. Focusing our analyses only on patients with concomitant ETA and BAL collection allowed us to precisely compare VAP incidences and describe microbial findings between different sampling methods. Thirdly, the low number of early VAP observed may have been influenced by the proportion of patients who received antibiotics just before ICU admission. Yet, we believe that this also represents a situation frequently observed during COVID-19 pandemic. Lastly, immunological findings are limited by many confounders (*e.g.*, the severity of COVID-19 disease, the use of steroids, and the length of ARDS), but we believe they can provide interesting glimpse for future analyses, not limited to COVID-19 patients but also for other viral pulmonary infections.

## Conclusions

In conclusion, in a cohort of prospectively enrolled mechanically ventilated COVID-19 patients, comparing distal to proximal sampling at VAP suspicion led to highly variable results in terms of VAP incidence and microbial findings. This should be considered when comparing VAP incidence between studies. Concordance between ETA and BAL was mainly limited by over 20% of ETA positive and BAL negative samples, while BAL molecular microbiology compared to conventional culture showed high sensitivity but limited specificity when evaluating in-panel targets only. Among the immunological markers examined at VAP suspicion, BAL IL-1β showed potential in discriminating microbiologically confirmed VAP.

### Supplementary Information

**Additional file 1**. Supplementary material.

## Data Availability

De-identified patient data used for the results reported in this article, including data in text, tables, figures, and appendices, will be available to researchers who provide a methodologically sound proposal to achieve their aims. Proposals should be addressed to emanuele.palomba@unimi.it and davide.mangioni@policlinico.mi.it. To gain access, data applicants will need to sign a data access agreement.
