# Supplementary material for: Incidence, microbiological and immunological characteristics of ventilator-associated pneumonia assessed by bronchoalveolar lavage and endotracheal aspirate in a prospective cohort of COVID-19 patients: CoV-AP study
Source: Crit Care. 2023 Sep 26;27:369. doi: 10.1186/s13054-023-04658-5 (PMC10521470; doi:10.1186/s13054-023-04658-5)
Supplement: Supplementary file 1 — Additional file 1. Supplementary material. [file 13054_2023_4658_MOESM1_ESM.doc]

**Incidence, microbiological and immunological characteristics of ventilator-associated pneumonia assessed by bronchoalveolar lavage and endotracheal aspirate in a prospective cohort of COVID-19 patients – CoV-AP study**

**SUPPLEMENTARY MATERIAL**

**METHODS**

*BAL acquisition*

Sampling was performed immediately after ETA recollection using disposable fiber optic bronchoscopes (Ambu® aScope™ 4 Broncho). The specific bronchial area to be sampled was determined based on the identification of lung consolidation on the chest X-ray. A total of five separate 20-mL portions of 0.9% NaCl at a temperature of 21 °C were administered. The objective was to collect at least 30% of the instilled volume during targeted sampling. The BAL samples were promptly transported to the laboratory for further processing.

*Microbiological analyses*

Bronchoscopy samples were analyzed for quantitative cultures (ETA and BAL) and BIOFIRE® FILMARRAY® Pneumonia Panel plus (BAL only). Briefly, 10 ul of bronchoscopy sample were cultured on *Haemophilus* chocolate 2 agar, Columbia agar + 5% sheep blood, Columbia ANC agar + 5% sheep blood  (incubated in 5% Co2 for 48 h at 36 ± 1°C), Mc Conkey agar, Mannitol salt 2 agar (incubated 48h at 36 ± 1°C) and Sabouraud agar (incubated for 5 days at 32 ± 1°C). All plates were supplied from Biomerieux. In parallel, the BioFire, FA-Pneumo assay was performed according to the manufacturer’s instructions from 200 μL sample.

*Immunological analyses*

Immunological analyses were performed within 24 hours from collection, on both whole peripheral blood (PB) and pre-filtered and concentrated BAL. Samples were stained with the MultitestTM 6-color TBNK Kit to evaluate lymphocyte subpopulations (absolute count or proportion) following the manufacturer’s instructions, and with a panel of monoclonal antibodies (CD14, CD16, HLA-DR, CD62L, CD4, CD8, CD15, CD3, CD45) with a lyse and wash procedure to determine monocytes and neutrophils subpopulations distribution, and the activation status of lymphocytes and monocytes.

PB were also tested for oxidative burst assay on neutrophils and monocytes according to Richardson et al.^1^. FACSLyric flow cytometer and FACSSuite software were used for acquisition and analysis. All reagents, instrument and software were from BD Biosciences.

Plasma samples were retrieved from PB leftovers and stored at -40°C until cytokines analysis were performed. Human Magnetic Luminex® custom assay (R&D Systems, Minneapolis, USA), LABScanTM 100 System and xPONENT software ver. 4.2 (Lagitre, Milan, Italy) were used to test the following analytes: IL-1α, IL-1β, IL-2, IL-6, IL-7, IL-10, IL-12 p70, CXCL10, MIP-1α and TNF-α.

#### Statistical Analysis

Descriptive statistics were produced for patients’ demographic, clinical and laboratory characteristics and any other variable of interest. Continuous variables were expressed as median together with the first and last quartile (Q1-Q3), and categorical variables were expressed as frequencies and proportions. Comparison between groups was made by applying T-Student test, Mann-Whitney test, Chi-squared test or Fisher’s exact test according to variable distribution. For all the analyses, tests were considered two-tailed with a significance level set at p<0.05. For immunological analyses, false discovery rate (FDR) method was considered to adjust the p-values obtained from multiple hypothesis tests conducted simultaneously. Cut-off value of BAL IL-1β to test the performance for VAP diagnosis was obtained using Youden Index of ROC curve.

Incidence rate (IR_VAP_) of first VAP event was calculated by dividing the number of patients who experienced VAP (according to ETA or BAL positivity) by the total MV-patient-day at risk (ventilator days), calculated from MV start to VAP diagnosis or MV end or death, whichever came first. To account for uncertainty, we calculated a 95% confidence interval of IR_VAP_ (95%CI) using a Poisson distribution.

Assuming as reference standard for VAP diagnosis the clinical criteria in combination with BAL analysis [3,4], we computed sensitivity, specificity, and positive and negative predictive values, including 95% confidence intervals (by Clopper Pearson exact method). Cohen’s kappa coefficient was used to assess the level of agreement between two diagnostic tests (ETA vs BAL and BAL_FAPPP_ vs BAL)^5,6^. Kappa p-value was calculated according to exact test due to the small sample size^7^. In case of very low or high cases distribution, PABAK was calculated to account for these factors^8,9^. Data were analyzed using the SAS software package, release 9.4 (SAS Institute, Cary, NC).

SUPPLEMENTARY MATERIAL – REFERENCES

1. Richardson MP, Ayliffe MJ, Helbert M, Davies EG. A simple flow cytometry assay using dihydrorhodamine for the measurement of the neutrophil respiratory burst in whole blood: comparison with the quantitative nitrobluetetrazolium test. *J Immunol Methods* 1998;219(1–2):187–193.

5. Cohen J. A Coefficient of Agreement for Nominal Scales. *Educ Psychol Meas* 1960;20(1):37–46.

6. Landis JR, Koch GG. The Measurement of Observer Agreement for Categorical Data. *Biometrics* 1977;33(1):159.

7. Gwet K. Computing inter-rater reliability with the SAS system. *Stat Methods Inter-rater Reliab Assess* 2002;3(3):1–16.

8. Feinstein AR, Cicchetti D V. High agreement but low Kappa: I. the problems of two paradoxes. *J Clin Epidemiol* 1990;43(6):543–549.

9. Byrt T, Bishop J, Carlin JB. Bias, prevalence and kappa. *J Clin Epidemiol* 1993;46(5):423–429.

**RESULTS**

**e-Figure 1**. Patient enrollment by month in the study period (from January 21, 2021 to May 2022)


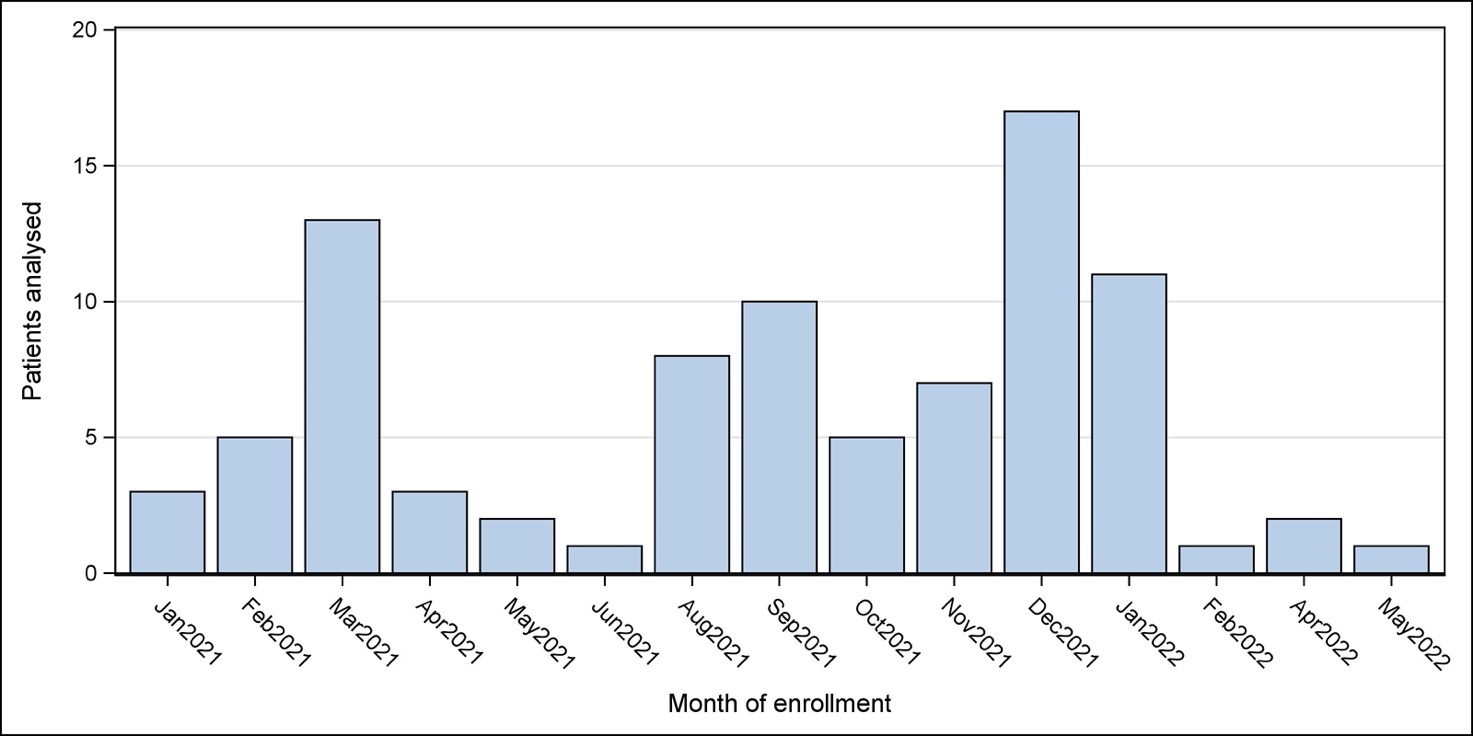


**e-Figure 2**. Immunological markers at VAP suspicion, according to sample site (peripheral blood or BAL) and microbial results (VAP or no-VAP by BAL). When feasible, blood/BAL ratio distribution was calculated. Immune cell markers are displayed in **Panel A**, selected cytokines in **Panel B**. In each box-plot, the horizontal line within the box represents the median, the box represents the interquartile range, the bottom edge of the box corresponds to the first quartile (Q1), marking the 25th percentile of the data, and the top edge represents the third quartile (Q3), marking the 75th percentile, the whiskers are the lines extending vertically from the box. The whiskers represent minimum and maximum values within a certain range, excluding outliers, individual data points that fall outside the whiskers are considered outliers and are represented by individual circles or x.


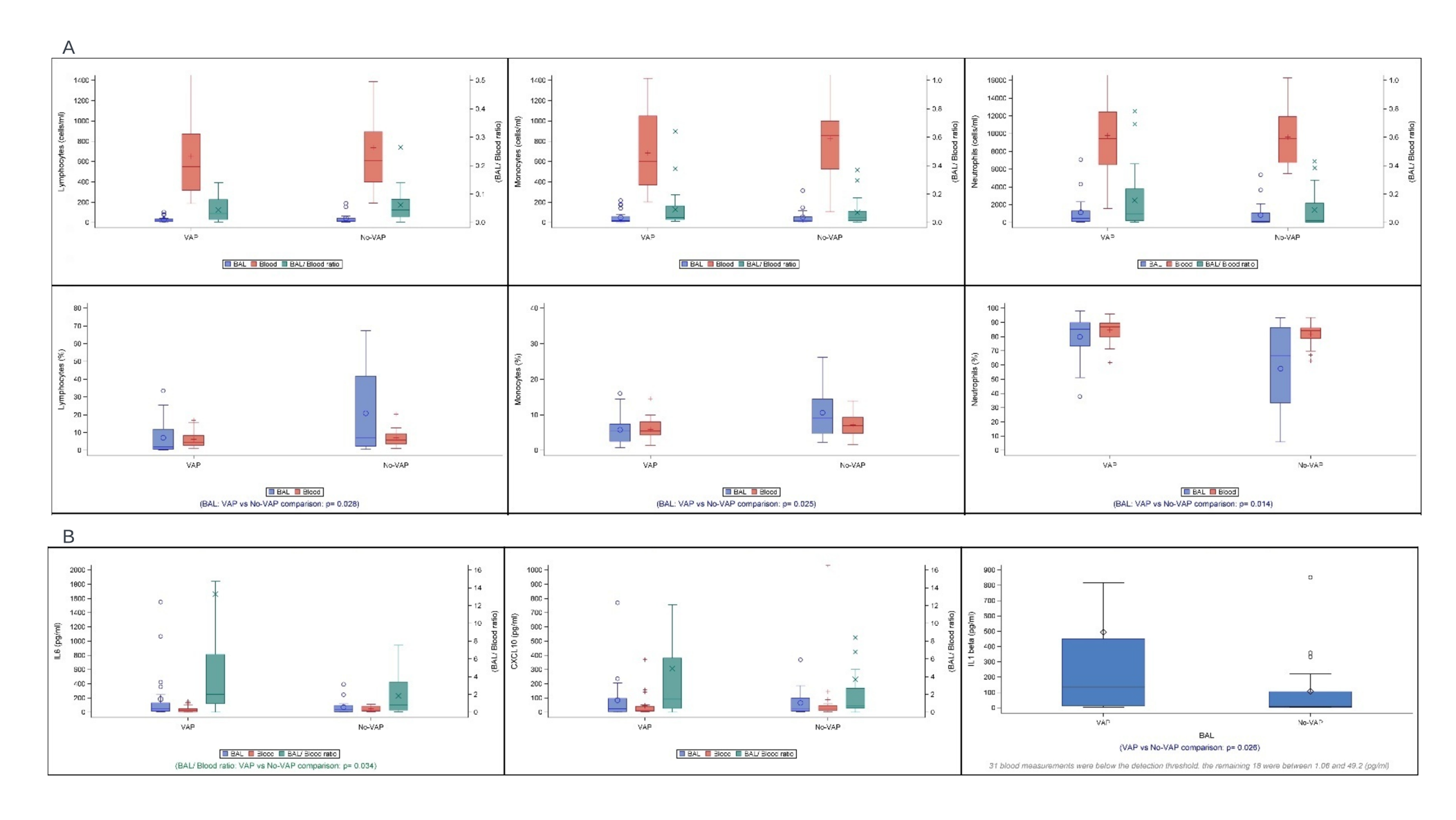


**e-Table 1**. Clinical characteristics and outcomes of the 90 patients enrolled with either ETA and/or BAL collected at VAP suspicion

|  | **Total**  **N = 90** | **Suspected VAP**  **N = 60** | **no-VAP suspicion**  **N = 30** |
| --- | --- | --- | --- |
| **Demographic characteristics** |  |  |  |
| Gender male | 64 (71.1) | 44 (73.3) | 20 (66.7) |
| Age, years | 60.0 (52.0-66.0) | 60.5 (52.5-66.0) | 58.5 (49.0-67.0) |
| BMI | 27.8 (25.0-31.2) | 27.7 (25.2-32.0) | 27.8 (24.6-30.1) |
| CCI |  |  |  |
| 0  1  2-3 | 62 (68.9)  20 (22.2)  8 (8.9) | 41 (68.3)  14 (23.3)  5 (8.3) | 21 (70.0)  6 (20.0)  3 (10.0) |
| **Clinical characteristics pre-ICU admission** |  |  |  |
| Days from symptoms onset to hospitalization | 6.0 (4.0-8.0) | 5.0 (3.0-8.0) | 6.0 (5.0-9.0) |
| Documented bacterial infections  CAP/HAP  Primary BSI/CRBSI  UTI | 10 (11.1)  3 (3.33)  3 (3.33)  4 (4.44) | 8 (13.3)  3 (5.0)  3 (5.0)  2 (3.3) | 2 (6.7)  0 (0.0)  0 (0.0)  2 (6.7) |
| Exposure to antibiotic therapy | 57 (63.3) | 34 (56.7) | 23 (76.7) |
| Exposure to steroid therapy  Standard^a^  High dose^b^  Both | 66 (73.3)  44 (48.9)  13 (14.4)  7 (7.8) | 43 (71.7)  28 (46.7)  8 (13.3)  5 (8.3) | 23 (76.7)  16 (53.3)  5 (16.7)  2 (6.7) |
| Exposure to other immunosuppressive therapy before admission^c^ | 14 (15.5) | 8 (13.3) | 6 (20.0) |
| **Clinical characteristics at ICU admission** |  |  |  |
| Days from hospitalization to MV start | 4.0 (1.0-7.0) | 4.0 (1.0-9.0) | 3.0 (1.0-5.0) |
| SOFA score | 6.0 (4.0-7.0) | 6.0 (4.0-7.5) | 6.0 (4.0-7.0) |
| PaO2:FiO2 ratio | 103.0 (76.0-120.0) | 103.5 (79.0-124.0) | 100.5 (74.0-120.0) |
| Leucocytes count, 10^3cell/*µ*L | 9.5 (7.3-13.4) | 9.8 (7.7-13.3) | 9.0 (7.1-14.7) |
| C reactive protein, mg/dl | 7.9 (4.1-17.7) | 7.3 (4.0-17.6) | 9.8 (4.6-19.9) |
| Procalcitonin, *µ*g/L | 0.2 (0.1-0.5) | 0.2 (0.1-0.5) | 0.2 (0.1-0.7) |
| **Clinical characteristics at VAP suspicion** |  |  |  |
| Days from MV start to VAP suspicion | - | 7.0 (5.0-9.5) | - |
| Days from last surveillance ETA to VAP suspicion | - | 2.0 (1.0-3.0) | - |
| Ongoing antibiotic therapy | - | 11 (18.3) | - |
| Ongoing steroid therapy  Standard^a^  High dose^b^ | - | 24 (40.0)  21 (35.0)  3 (5.0) | - |
| Prone position | - | 29 (48.3) | - |
| ECMO support | - | 4 (6.7) | - |
| SOFA score | - | 5.0 (3.5- 6.0) | - |
| PaO2:FiO2 ratio | - | 136.0 (106.2-160.9) | - |
| Leucocytes count, 10^3cell/*µ*L | - | 10.9 (8.4-14.0) | - |
| C reactive protein, mg/dl | - | 16.0 (11.3-19.9) | - |
| Procalcitonin, *µ*g/L | - | 0.3 (0.2-0.6) | - |
| MR-proADM nmol/L | - | 1.2 (0.9-1.6) | - |
| Secondary BSI | - | 13 (21.7) | - |
| **Outcome characteristics** |  |  |  |
| MV duration, days* | 17.5 (10.0-30.0) | 23.5 (15.0-39.0) | 7.5 (5.0-13.0) |
| ICU length of stay, days* | 19.0 (10.0-32.0) | 25.5 (16.0-42.5) | 9.0 (6.0-15.0) |
| ICU mortality** | 30 (33.3) | 24 (40.0) | 6 (20.0) |

Legend: VAP ventilator associated pneumonia, BMI body mass index, CCI charlson comorbidity index (age-unadjusted), ICU intensive care unit, CAP community-acquired pneumonia, HAP hospital-associated pneumonia, BSI bloodstream infection, CRBSI catheter-related bloodstream infection, UTI urinary tract infection, MV mechanical ventilation, VAP ventilator associated pneumonia, ECMO extracorporeal membrane oxygenation, SOFA score sequential [sepsis-related] organ failure assessment score. ^a^Standard: dexamethasone (any dosage) or methylprednisolone ≤1mg/kg/day; ^b^High dose: methylprednisolone >1mg/kg/day; ^c^among patients with no-VAP suspicion, 3 (10.0%) assumed Barnicitinib and 3 (10.0%) assumed Tocilizumab; among patients with VAP suspicion, 4 (6.7%) assumed Barnicitinib and 4 (6.7%) assumed Barnicitinib; *Mann-Whitney U test p-value < 0.001; **Chi-square test p-value = 0.058

**e-Table 2**. Clinical characteristics and outcomes of the 49 patients enrolled with suspected VAP, overall and stratified according to ETA results

|  | **Total**  **N = 49** | **ETA pos**  **N = 35** | **ETA neg**  **N = 14** |
| --- | --- | --- | --- |
| **Demographic characteristics** |  |  |  |
| Gender male | 35 (71.4) | 24 (68.6) | 11 (78.6) |
| Age, years | 61.0 (53.0-66.0) | 61.0 (53.0-69.0) | 60.5 (46.0-63.0) |
| BMI | 27.7 (25.2-32.7) | 27.1 (24.8-31.2) | 29.0 (27.5-33.9) |
| CCI |  |  |  |
| 0  1  2-3 | 33 (67.4)  11 (22.4)  5 (10.2) | 22 (62.9)  9 (25.7)  4 (11.4) | 11 (78.6)  2 (14.3)  1 (7.1) |
| **Clinical characteristics pre-ICU admission** |  |  |  |
| Days from symptoms onset to hospitalization | 5.0 (3.0-7.0) | 5.0 (3.0-8.0) | 7.0 (5.0 -7.0) |
| Documented bacterial infections  CAP/HAP  Primary BSI/CRBSI  UTI | 7 (14.3)  3 (6.1)  2 (4.1)  2 (4.1) | 3 (8.6)  1 (2.9)  1 (2.9)  1 (2.9) | 4 (28.6)  2 (14.3)  1 (14.3)  1 (14.3) |
| Exposure to antibiotic therapy | 28 (57.1) | 18 (51.4) | 10 (71.4) |
| Exposure to steroid therapy  Standard^a^  High dose^b^  Both | 37 (75.5)  23 (46.9)  7 (14.3)  5 (10.2) | 26 (74.3)  16 (45.7)  5 (14.3)  4 (11.4) | 11 (78.6)  7 (50.0)  2 (14.3)  1 (7.1) |
| Exposure to other immunosuppressive therapy before admission | 6 (12.2) | 2 (5.7) | 4 (28.6) |
| **Clinical characteristics at ICU admission** |  |  |  |
| Days from hospitalization to MV start | 4.0 (3.0-9.0) | 4.0 (1.0-7.0) | 7.5 (4.0-11.0) |
| SOFA score | 6.0 (4.0-8.0) | 5.0 (4.0-8.0) | 6.0 (5.0-8.0) |
| PaO2:FiO2 ratio | 102.0 (73.0-124.0) | 103.0 (79.0-124.0) | 88.0 (58.0-124.0) |
| Leucocytes count, 10^3cell/*µ*L | 9.9 (7.9-13.3) | 9.3 (7.6-12.5) | 11.5 (9.9-13.9) |
| C reactive protein, mg/dl | 7.0 (3.9-17.5) | 6.9 (3.3-13.3) | 10.4 (4.6-18.1) |
| Procalcitonin, *µ*g/L | 0.2 (0.1-0.5) | 0.2 (0.1-0.6) | 0.2 (0.1-0.3) |
| **Clinical characteristics at VAP suspicion** |  |  |  |
| Days from MV start to VAP suspicion | 6.0 (5.0-9.0) | 6.0 (5.0-9.0) | 6.5 (6.0-11.0) |
| Days from last surveillance ETA to VAP suspicion | 1.5 (1.0-3.0) | 1.5 (1.0-3.0) | 1.5 (1.0-2.0) |
| Ongoing antibiotic therapy | 9 (18.4) | 6 (17.1) | 3 (21.4) |
| Ongoing steroid therapy*  Standard^a^*  High dose^b^ | 20 (40.8)  17 (34.7)  3 (6.1) | 18 (51.4)  16 (45.7)  2 (5.7) | 2 (14.3)  1(7.14)  1 (7.14) |
| Prone position | 23 (46.9) | 16 (45.7) | 7 (50.0) |
| ECMO support | 3 (6.1) | 1 (2.9) | 2 (14.4) |
| SOFA score | 5.0 (3.0-6.0) | 5.0 (3.0-6.0) | 6.0 (4.0-7.0) |
| PaO2:FiO2 ratio | 136.0 (108.6-162.2) | 140.0 (113.8-162.2) | 128.0 (91.8-163.3) |
| Leucocytes count, 10^3cell/*µ*L | 11.0 (8.5-14.3) | 10.5 (7.6-13.4) | 11.3 (10.1-15.4) |
| C reactive protein, mg/dl | 16.0 (10.9-20.0) | 14.5 (10.0-20.6) | 16.6 (13.1-20.0) |
| Procalcitonin, *µ*g/L | 0.3 (0.2-0.6) | 0.3 (0.2-0.9) | 0.3 (0.1-0.5) |
| MR-proADM nmol/L | 1.2 (0.9-1.6) | 1.3 (0.9-1.6) | 1.1 (1.0-1.5) |
| Secondary BSI | 9 (18.4) | 8 (22.8) | 1 (7.1) |
| **Outcome characteristics** |  |  |  |
| MV duration, days | 24.0 (16.0-44.0) | 24.0 (16.0-44.0) | 25.5 (18.0-51.0) |
| ICU length of stay, days | 27.0 (17.0-55.0) | 26.0 (16.0-48.0) | 31.0 (18.0-55.0) |
| ICU mortality | 20 (40.8) | 16 (45.7) | 4 (28.6) |

Legend: BMI body mass index, CCI charlson comorbidity index (age-unadjusted), ICU intensive care unit, CAP community-acquired pneumonia, HAP hospital-associated pneumonia, BSI bloodstream infection, CRBSI catheter-related bloodstream infection, UTI urinary tract infection, MV mechanical ventilation, VAP ventilator associated pneumonia, ECMO extracorporeal membrane oxygenation, SOFA score sequential [sepsis-related] organ failure assessment score. ^a^Standard: dexamethasone (any dosage) or methylprednisolone ≤1mg/kg/day; ^b^High dose: methylprednisolone >1mg/kg/day; * Fisher exact test p-value=0.02

**e-Table 3**. Clinical characteristics and outcomes of the 49 patients enrolled with suspected VAP, overall and stratified according to BAL results

|  | **Total**  **N = 49** | **BAL pos**  **N = 27** | **BAL neg**  **N = 22** |
| --- | --- | --- | --- |
| **Demographic characteristics** |  |  |  |
| Gender male | 35 (71.4) | 17 (63.0) | 18 (81.8) |
| Age, years | 61.0 (53.0-66.0) | 65.0 (57.0-70.0) | 57.0 (51.0-63.0) |
| BMI | 27.7 (25.2-32.7) | 27.7 (24.8-33.9) | 27.7 (25.4-31.2) |
| CCI |  |  |  |
| 0  1  2-3 | 33 (67.4)  11 (22.4)  5 (10.2) | 15 (55.6)  9 (33.3)  3 (11.1) | 18 (81.8)  2 (9.1)  2 (9.1) |
| **Clinical characteristics pre-ICU admission** |  |  |  |
| Days from symptoms onset to hospitalization | 5.0 (3.0-7.0) | 5.0 (3.0-7.0) | 7.0 (5.0-9.0) |
| Documented bacterial infections  CAP/HAP  Primary BSI/CRBSI  UTI | 7 (14.3)  3 (6.1)  2 (4.1)  2 (4.1) | 3 (11.1)  2 (7.4)  0 (0.0)  1 (3.7) | 4 (18.2)  1 (4.6)  2 (9.1)  1 (4.6) |
| Exposure to antibiotic therapy | 28 (57.1) | 13 (48.2) | 15 (68.2) |
| Exposure to steroid therapy  Standard^a^  High dose^b^  Both | 37 (75.5)  23 (46.9)  7 (14.3)  5 (10.2) | 19 (70.4)  13 (48.2)  3 (11.1)  2 (7.4) | 18 (81.8)  10 (45.5)  4 (18.2)  3 (13.6) |
| Exposure to other immunosuppressive therapy before admission | 6 (12.2) | 3 (11.1) | 3 (13.6) |
| **Clinical characteristics at ICU admission** |  |  |  |
| Days from hospitalization to MV start* | 4.0 (3.0-9.0) | 3.0 (1.0-6.0) | 6.5 (4.0-11.0) |
| SOFA score | 6.0 (4.0-8.0) | 6.0 (4.0-8.0) | 6.0 (4.0-8.0) |
| PaO2:FiO2 ratio | 102.0 (73.0-124.0) | 103.0 (73.0-135.0) | 95.5 (73.0-120.0) |
| Leucocytes count, 10^3cell/*µ*L | 9.9 (7.9-13.3) | 9.5 (7.8-12.6) | 10.6 (7.9-13.4) |
| C reactive protein, mg/dl | 7.0 (3.9-17.5) | 10.3 (5.2-19.5) | 5.2 (2.2-12.6) |
| Procalcitonin, *µ*g/L | 0.2 (0.1-0.5) | 0.2 (0.1-0.6) | 0.2 (0.1-0.4) |
| **Clinical characteristics at VAP suspicion** |  |  |  |
| Days from MV start to VAP suspicion** | 6.0 (5.0-9.0) | 5.0 (4.0-7.0) | 9.0 (6.0-11.0) |
| Days from last surveillance ETA to VAP suspicion | 1.5 (1.0-3.0) | 2.0 (1.0-3.0) | 1.0 (1.0-3.0) |
| Ongoing antibiotic therapy | 9 (18.4) | 5 (18.5) | 4 (18.2) |
| Ongoing steroid therapy***  Standard^a^***  High dose^b^ | 20 (40.8)  17 (34.7)  3 (6.1) | 16 (59.3)  14 (51.8)  2 (7.4) | 4 (18.2)  3 (13.6)  1 (4.6) |
| Prone position | 23 (46.9) | 13 (48.2) | 10 (45.5) |
| ECMO support | 3 (6.1) | 1 (3.7) | 2 (9.1) |
| SOFA score | 5.0 (3.0-6.0) | 5.0 (3.0-6.0) | 5.0 (4.0-6.0) |
| PaO2:FiO2 ratio | 136.0 (108.6-162.2) | 140.0 (108.6-162.2) | 131.3 (106.2-163.3) |
| Leucocytes count, 10^3cell/*µ*L | 11.0 (8.5-14.3) | 11.5 (8.3-14.6) | 10.4 (8.5-13.4) |
| C reactive protein, mg/dl | 16.0 (10.9-20.0) | 16.1 (10.9-20.6) | 16.0 (10.0-20.0) |
| Procalcitonin, *µ*g/L | 0.3 (0.2-0.6) | 0.3 (02-0.6) | 0.3 (0.2-0.7) |
| MR-proADM nmol/L | 1.2 (0.9-1.6) | 1.4 (0.8-2.0) | 1.1 (1.0-1.5) |
| Secondary BSI | 9 (18.4) | 7 (25.9) | 2 (9.1) |
| **Outcome characteristics** |  |  |  |
| MV duration, days | 24.0 (16.0-44.0) | 24.0 (15.0-35.0) | 31.0 (20.0-57.0) |
| ICU length of stay, days | 27.0 (17.0-55.0) | 25.0 (15.0-36.0) | 38.0 (21.0-61.0) |
| ICU mortality | 20 (40.8) | 14 (51.8) | 6 (27.3) |

Legend: BMI body mass index, CCI charlson comorbidity index (age-unadjusted), ICU intensive care unit, CAP community-acquired pneumonia, HAP hospital-associated pneumonia, BSI bloodstream infection, CRBSI catheter-related bloodstream infection, UTI urinary tract infection, MV mechanical ventilation, VAP ventilator associated pneumonia, ECMO extracorporeal membrane oxygenation, SOFA score sequential [sepsis-related] organ failure assessment score. ^a^Standard: dexamethasone (any dosage) or methylprednisolone ≤1mg/kg/day; ^b^High dose: methylprednisolone >1mg/kg/day; *Mann-Whitney U test p-value=0.01; **Mann-Whitney U test p-value<0.01; ***Fisher exact test p-value=0.01

**e-Table 4**. Clinical characteristics at VAP suspicion of the 49 patients enrolled with both ETA and BAL collection, overall and stratified according to combinations of ETA and BAL results

|  | **Total**  **N = 49** | **ETA+/BAL+**  **N = 24** | **ETA+/BAL-**  **N = 11** | **ETA-/BAL+**  **N = 3** | **ETA-/BAL-**  **N = 11** |
| --- | --- | --- | --- | --- | --- |
| Days from MV start to VAP suspicion | 6.0 (5.0-9.0) | 5.0 (4.0-7.0) | 9.0 (7.0-13.0) | 6.0 (5.0-62.0) | 7.0 (6.0-11.0) |
| Days from last surveillance ETA to VAP suspicion | 1.5 (1.0-3.0) | 2.0 (1.0-3.0) | 1.0 (1.0-3.0) | 2.0 (1.0-2.0) | 1.0 (1.0-3.0) |
| Ongoing antibiotic therapy | 9 (18.4) | 4 (16.7) | 2 (18.2) | 1 (33.3) | 2 (18.2) |
| Ongoing steroid therapy  Standard^a^  High dose^b^ | 20 (40.8)  17 (34.7)  3 (6.1) | 15 (62.5)  14 (58.3)  1 (4.2) | 3 (27.3)  2 (18.2)  1 (9.01) | 1 (33.3)  0 (0.0)  1 (33.3) | 1 (9.1)  1 (9.1)  0 (0.0) |
| Prone position | 23 (46.9) | 11 (45.8) | 5(45.5) | 2 (66.7) | 5 (45.5) |
| ECMO support | 3 (6.1) | 0 (0.0) | 1 (9.01) | 1 (33.3) | 1 (9.1) |
| SOFA score | 5.0 (3.0-6.0) | 5.0 (3.0-6.0) | 4.0 (3.0-6.0) | 7.0 (6.0-7.0) | 5.0 (4.0-7.0) |
| PaO2:FiO2 ratio | 136.0 (108.6-162.2) | 142  (120-161) | 127  (113-184) | 109  (75-178) | 136  (92-163) |
| Leucocytes count, 10^3cell/µL | 11.0 (8.5-14.3) | 11.3  (7.9-14.5) | 9.5  (7.6-12.1) | 13.2  (11.3-21.2) | 10.8  (8.5-15.4) |
| C reactive protein, mg/dl | 16.0 (10.9-20.0) | 15.1  (11.4-19.7) | 14.5  (6.1-29.1) | 16.1  (10.9-23.2) | 17.0  (13.1-20.0) |
| Procalcitonin, µg/L | 0.3 (0.2-0.6) | 0.3 (0.2-0.8) | 0.3 (0.2-2.9) | 0.3 (0.1-0.5) | 0.3 (0.1-0.7) |
| MR-proADM nmol/L | 1.2 (0.9-1.6) | 1.4 (0.8-2.0) | 1.1 (0.9-1.6) | 1.4 (0.7-2.1) | 1.1 (1.0-1.5) |
| Secondary BSI | 9 (18.4) | 6 (25.0) | 2 (18.2) | 1 (33.3) | 0 (0.0) |

Legend: MV mechanical ventilation, VAP ventilator associated pneumonia, ECMO extracorporeal membrane oxygenation, SOFA score sequential [sepsis-related] organ failure assessment score. ^a^Standard: dexamethasone (any dosage) or methylprednisolone ≤1mg/kg/day; ^b^High dose: methylprednisolone >1mg/kg/day

**e-Table 5**. Pathogens isolated in microbiologically confirmed VAP of patients enrolled with concomitant ETA and BAL collection at VAP suspicion (n° 49). Microbial species are reported according to different respiratory samples and diagnostic techniques.

|  | **ETA** | **BAL** | **BAL_FAPPP_** |
| --- | --- | --- | --- |
| Number of positive samples | 35 | 27 | 28 |
| Polymicrobial isolates | 9/35 (25.7) | 7/27 (25.9) | 11/28 (39.2) |
| Total number of microbial isolates | 46 | 34 | 41 |
| **Gram positive** | **14 (30.4)** | **13 (38.2)** | **15 (36.6)** |
| *Staphylococcus aureus* | 10 (21.7) | 8 (23.5) | 13 (31.7) |
| *Streptococcus agalatiae* | 0 | 0 | 1 (2.4) |
| *Streptococcus pneumoniae* | 0 | 1 (2.9) | 1 (2.4) |
| *Corynebacterium striatum* | 4 (8.7) | 4 (11.8) | - |
| **Gram negative** | **26 (56.5)** | **17 (50.0)** | **26 (63.4)** |
| *Klebsiella pneumoniae* | 2 (4.3) | 3 (8.8) | 3 (7.32) |
| *Klebsiella* spp | 4 (8.7) | 3 (8.8) | 2 (4.9) |
| *Escherichia coli* | 2 (4.3) | 2 (5.9) | 4 (9.8) |
| *Proteus* spp | 1 (2.2) | 0 | 2 (4.9) |
| *Enterobacter* spp | 2 (4.3) | 0 | 1 (2.4) |
| *Hafnia alvei* | 2 (4.3) | 1 (2.9) | - |
| *Serratia marcescens* | 2 (4.3) | 1 (2.9) | 2 (4.9) |
| *Haemophilus influenzae* | 1 (2.2) | 1 (2.9) | 3 (7.32) |
| *Pseudomonas aeruginosa* | 9 (19.6) | 5 (14.7) | 8 (19.5) |
| *Acinetobacter baumannii* | 1 (2.2) | 1 (2.9) | 1 (2.4) |
| **Fungi (molds)** | **6 (13.0)** | **4 (11.8)** | **-** |
| *Aspergillus* spp | 6 (13.0) | 4 (11.8) | - |

Legend: ETA endotracheal aspirate - conventional culture, BAL bronchialveolar lavage - conventional culture, BAL_FAPPP_ bronchoalveolar lavage – molecular diagnostics (Filmarray Pneumonia Panel Plus), MDR multidrug resistant, *Klebsiella* spp *Klebsiella* species other than *K. pneumoniae* and *K. aerogenes*, *Enterobacter* spp including *Klebsiella* (*Enterobacter*) *aerogenes* due to similar resistance mechanism

**e-Table 6**. Pathogens isolated in microbiologically confirmed VAP of patients enrolled with ETA and/or BAL collection at VAP suspicion (n° 60). Microbial species are reported according to different respiratory samples and diagnostic techniques.

|  | **ETA** | **BAL** | **BAL_FAPPP_** |
| --- | --- | --- | --- |
| Number of positive samples | 44/59 | 28/50 | 31/52 |
| Polymicrobial isolates | 13/44 (29.5) | 7/28 (25.0) | 12/31 (38.7) |
| Total number of microbial isolates | 60 | 35 | 45 |
| **Gram positive** | **21 (35.0)** | **13 (37.1)** | **17 (37.8)** |
| *Staphylococcus aureus* | 17 (28.3) | 8 (22.8) | 15 (33.3) |
| *Streptococcus agalatiae* | 0 | 0 | 1 (2.2) |
| *Streptococcus pneumoniae* | 0 | 1 (2.9) | 1 (2.2) |
| *Corynebacterium striatum* | 4 (6.7) | 4 (11.4) | - |
| **Gram negative** | **31 (51.7)** | **18 (51.4)** | **28 (62.2)** |
| *Klebsiella pneumoniae* | 4 (6.7) | 3 (8.6) | 4 (8.9) |
| *Klebsiella* spp | 5 (8.3) | 3 (8.6) | 2 (4.4) |
| *Escherichia coli* | 2 (3.3) | 2 (5.7) | 4 (8.9) |
| *Proteus* spp | 1 (1.7) | 0 | 2 (4.4) |
| *Enterobacter* spp | 3 (5.0) | 0 | 1 (2.2) |
| *Hafnia alvei* | 2 (3.3) | 1 (2.9) | - |
| *Serratia marcescens* | 2 (3.3) | 1 (2.9) | 2 (4.4) |
| *Haemophilus influenzae* | 1 (1.7) | 1 (2.9) | 3 (6.7) |
| *Pseudomonas aeruginosa* | 10 (16.7) | 5 (14.3) | 9 (20.0) |
| *Acinetobacter baumannii* | 1 (1.7) | 1 (2.9) | 1 (2.2) |
| *Burkholderia gladioli* | 0 | 1 (2.9) | - |
| **Fungi (molds)** | **8 (13.3)** | **4 (11.4)** | **-** |
| *Aspergillus* spp | 8 (13.3) | 4 (11.4) | - |

Legend: ETA endotracheal aspirate - conventional culture, BAL bronchialveolar lavage - conventional culture, BAL_FAPPP_ bronchoalveolar lavage – molecular diagnostics (Filmarray Pneumonia Panel Plus), MDR multidrug resistant, *Klebsiella* spp *Klebsiella* species other than *K. pneumoniae* and *K. aerogenes*, *Enterobacter* spp including *Klebsiella* (*Enterobacter*) *aerogenes* due to similar resistance mechanism

**e-Table 7.** Secondary BSI in patients enrolled with concomitant ETA and BAL collection at VAP suspicion (n° 49).

Microbial species, bacterial load (for respiratory samples), and days between bronchoscopic sampling and positive blood cultures are reported.

| **CoV-AP patient ID** | **ETA_microbial species and bacterial load** | **BAL_ microbial species and bacterial load** | **BALFAPPP_ microbial species and bacterial load** | **sBSI* (YES/NO)** | **sBSI_ microbial species** | **sBSI_time from VAP, days** |
| --- | --- | --- | --- | --- | --- | --- |
| **COVAP 001** | Corynebacterium striatum 10^6 | Corynebacterium striatum 10^4 | neg | NO |  |  |
| **COVAP 003** | Pseudomonas aeruginosa 10^5 | neg | Pseudomonas aeruginosa 10^5 | YES | Pseudomonas aeruginosa | -1 |
| **COVAP 004** | Pseudomonas aeruginosa 10^6 | Pseudomonas aeruginosa 10^6 | Pseudomonas aeruginosa 10^7 | YES | Pseudomonas aeruginosa | 5 |
| **COVAP 005** | neg | neg | neg | / |  |  |
| **COVAP 009** | neg | neg | neg | / |  |  |
| **COVAP 011** | Hafnia alvei 10^5 | Aspergillus fumigatus | neg | NO |  |  |
| **COVAP 012** | Aspergillus fumigatus | neg | neg | NO |  |  |
| **COVAP 015** | Corynebacterium striatum 10^6 | Corynebacterium striatum 10^4 | neg | NO |  |  |
| **COVAP 016** | neg | Klebsiella pneumoniae 10^4 | Klebsiella pneumoniae 10^6 | NO |  |  |
| **COVAP 017** | neg | neg | neg | / |  |  |
| **COVAP 018** | Pseudomonas aeruginosa 10^6 | Pseudomonas aeruginosa 10^5 | Pseudomonas aeruginosa 10^6 | NO |  |  |
| **COVAP 019** | Aspergillus fumigatus | Aspergillus fumigatus | neg | NO |  |  |
| **COVAP 020** | Pseudomonas aeruginosa 10^6, Enterobacter hormaechei 10^5 | neg | Pseudomonas aeruginosa 10^4 | NO |  |  |
| **COVAP 023** | Staphylococcus aureus 10^5 | neg | Staphylococcus aureus 10^4 | NO |  |  |
| **COVAP 024** | Staphylococcus aureus 10^5 | Staphylococcus aureus 10^4 | Staphylococcus aureus 10^6, Proteus spp 10^4 | YES | Staphylococcus aureus | 5 |
| **COVAP 025** | Corynebacterium striatum 10^6 | Corynebacterium striatum 10^6 | neg | NO |  |  |
| **COVAP 026** | Serratia marcescens 10^5 | neg | Serratia marcescens 10^6 | NO |  |  |
| **COVAP 027** | Acinetobacter baumannii >10^6, Klebsiella pneumoniae >10^6 | Acinetobacter baumannii >10^6, Klebsiella pneumoniae >10^6 | Acinetobacter baumannii 10^6, Klebsiella pneumoniae 10^5, Streptococcus agalactiae 10^6 | NO |  |  |
| **COVAP 029** | Staphylococcus aureus 10^6 | neg | Staphylococcus aureus 10^5 | NO |  |  |
| **COVAP 030** | Aspergillus spp | neg | neg | NO |  |  |
| **COVAP 032** | Pseudomonas aeruginosa 10^6 | Pseudomonas aeruginosa 10^6 | Pseudomonas aeruginosa 10^7 | NO |  |  |
| **COVAP 031** | Staphylococcus aureus >10^6, Klebsiella aerogenes >10^6 | Staphylococcus aureus 10^4 | Staphylococcus aureus 10^5 | YES | Staphylococcus aureus | 1 |
| **COVAP 036** | Corynebacterium striatum 10^6 | Corynebacterium striatum >10^6 | neg | NO |  |  |
| **COVAP 039** | neg | neg | neg | / |  |  |
| **COVAP 037** | neg | neg | neg | / |  |  |
| **COVAP 044** | Klebsiella aerogenes >10^6, Pseudomonas aeruginosa >10^6 | Klebsiella aerogenes 10^6 | neg | YES | Klebsiella aerogenes | 13 |
| **COVAP 038** | neg | neg | Escherichia coli 10^5, Haemophilus influenzae 10^6 | (NO) |  |  |
| **COVAP 045** | Enterobacter cloacae >10^6, Staphylococcus aureus >10^6, Aspergillus spp | Aspergillus spp, Staphylococcus aureus 10^6 | Enterobacter cloacae 10^5, Staphylococcus aureus >10^7 | NO |  |  |
| **COVAP 046** | Pseudomonas aeruginosa >10^6 | Pseudomonas aeruginosa >10^6 | Haemophilus influenzae 10^6, Pseudomonas aeruginosa >10^7 | NO |  |  |
| **COVAP 053** | Staphylococcus aureus 10^5 | Staphylococcus aureus 10^5 | Staphylococcus aureus > 10^7 | NO |  |  |
| **COVAP 055** | Staphylococcus aureus > 10^6; Escherichia coli > 10^6 | Staphylococcus aureus > 10^6; Escherichia coli > 10^6 | Staphylococcus aureus > 10^7, Escherichia coli > 10^7 | NO |  |  |
| **COVAP 056** | Klebsiella pneumoniae 10^5 | Klebsiella pneumoniae 10^4 | Klebsiella pneumoniae 10^6 | NO |  |  |
| **COVAP 058** | neg | neg | neg | / |  |  |
| **COVAP 061** | neg | Aspergillus fumigatus | neg | NO |  |  |
| **COVAP 064** | Klebsiella aerogenes > 10^6 | Klebsiella aerogenes > 10^6 | Klebsiella aerogenes > 10^7 | NO |  |  |
| **COVAP 065** | Klebsiella aerogenes 10^5; Pseudomonas aeruginosa 10^5 | Klebsiella aerogenes 10^5; Pseudomonas aeruginosa 10^5 | Klebsiella aerogenes 10^5; Pseudomonas aeruginosa 10^5 | NO |  |  |
| **COVAP 067** | Haemophilus influenzae > 10^6; Hafnia alvei > 10^6 | Haemophilus influenzae > 10^6; Hafnia alvei 10^5 | Haemophilus influenzae > 10^7 | NO |  |  |
| **COVAP 071** | Serratia marcescens >10^6 | Staphylococcus aureus 10^4; Serratia marcescens > 10^6 | Proteus spp 10^5; Serratia marcescens > 10^7; Staphylococcus aureus 10^5 | YES | Staphylococcus aureus | 2 |
| **COVAP 072** | Aspergillus fumigatus group | neg | neg | NO |  |  |
| **COVAP 073** | neg | neg | neg | / |  |  |
| **COVAP 076** | Staphylococcus aureus 10^6 | Staphylococcus aureus 10^4 | Staphylococcus aureus > 10^7 | YES | Staphylococcus aureus | -2 |
| **COVAP 082** | Pseudomonas aeruginosa 10^6 | neg | Pseudomonas aeruginosa 10^6 | NO |  |  |
| **COVAP 084** | Staphylococcus aureus 10^6; Escherichia coli > 10^6; Aspergillus fumigatus | Staphylococcus aureus 10^5; Escherichia coli > 10^6 | Staphylococcus aureus > 10^7; Escherichia coli > 10^7 | YES | Staphylococcus aureus, Escherichia coli | 0 |
| **COVAP 085** | neg | neg | neg | / |  |  |
| **COVAP 087** | neg | Streptococcus pneumoniae 10^6 | Staphylococcus aureus 10^6; Streptococcus pneumoniae > 10^7 | NO |  |  |
| **COVAP 088** | Staphylococcus aureus >10^6 | neg | Escherichia coli 10^7; Staphylococcus aureus 10^7 | YES | Staphylococcus aureus | 2 |
| **COVAP 089** | neg | neg | neg | / |  |  |
| **COVAP 093** | neg | neg | Staphylococcus aureus > 10^7 | (NO) |  |  |
| **COVAP 094** | Proteus mirabilis 10^5 | neg | neg | NO |  |  |

*sBSI is defined according to the Center of Diseases Control and Prevention (CDC) National Healthcare Safety Network (NHSN) Patient Safety Component Manual, version January 2021. This requires the isolation on blood cultures of the same organism identified from ETA and/or BAL within the secondary BSI attribution period, that includes the infection window period (+/- 3 days from the date of event) and the repeat infection timeframe (+14 days from the date of event).

**e-Table 8**. Microbial concordance of ETA vs BAL in patients enrolled with concomitant ETA and BAL collection at VAP suspicion (n° 49).

| ETA | BAL | Shared species/ species identified | Patients  N (%) |
| --- | --- | --- | --- |
| **Complete species concordance** |  |  | **18 (36.7)** |
| *Corynebacterium striatum* | *Corynebacterium striatum* | 1/1 | 4 |
| *Pseudomonas aeruginosa* | *Pseudomonas aeruginosa* | 1/1 | 4 |
| *Staphylococcus aureus* | *Staphylococcus aureus* | 1/1 | 3 |
| *Klebsiella pneumoniae* | *Klebsiella pneumoniae* | 1/1 | 1 |
| *Klebsiella* spp | *Klebsiella* spp | 1/1 | 1 |
| *Aspergillus* spp | *Aspergillus* spp | 1/1 | 1 |
| *Acinetobacter baumannii* complex*/ Klebsiella pneumoniae* | *Acinetobacter baumannii complex/ Klebsiella pneumoniae* | 2/2 | 1 |
| *Haemophilus influenzae/Hafnia alvei* | *Haemophilus influenzae/Hafnia alvei* | 2/2 | 1 |
| *Klebsiella* spp*/ Pseudomonas aeruginosa* | *Klebsiella* spp*/ Pseudomonas aeruginosa* | 2/2 | 1 |
| *Staphylococcus aureus/ Escherichia coli* | *Staphylococcus aureus/ Escherichia coli* | 2/2 | 1 |
| **Partial species concordance** |  |  | **5 (10.2)** |
| *Serratia marcescens* | *Serratia marcescens / Staphylococcus aureus* | 1/2 | 1 |
| *Klebsiella* spp */ Pseudomonas aeruginosa* | *Klebsiella* spp | 1/2 | 1 |
| *Staphylococcus aureus / Klebsiella* spp | *Staphylococcus aureus* | 1/2 | 1 |
| *Enterobacter* spp */ Staphylococcus aureus / Aspergillus* spp | *Staphylococcus aureus / Aspergillus* spp | 2/3 | 1 |
| *Staphylococcus aureus / Escherichia coli / Aspergillus* spp | *Staphylococcus aureus / Escherichia coli* | 2/3 | 1 |
| **Total species discordance** |  |  | **15 (30.6)** |
| Negative | *Aspergillus* spp | 0/1 | 1 |
| Negative | *Klebsiella pneumoniae* | 0/1 | 1 |
| Negative | *Streptococcus pneumoniae* | 0/1 | 1 |
| *Aspergillus* spp | Negative | 0/1 | 3 |
| *Proteus mirabilis* | Negative | 0/1 | 1 |
| *Pseudomonas aeruginosa* | Negative | 0/1 | 2 |
| *Serratia marcescens* | Negative | 0/1 | 1 |
| *Staphylococcus aureus* | Negative | 0/1 | 3 |
| *Hafnia alvei* | *Aspergillus* spp | 0/2 | 1 |
| *Enterobacter* spp */ Pseudomonas aeruginosa* | Negative | 0/2 | 1 |
| **Both negative samples (VAP suspicion not confirmed)** |  | | **11 (22.4)** |

Legend: ETA endotracheal aspirate - conventional culture, BAL bronchialveolar lavage - conventional culture, *Klebsiella* spp *Klebsiella* species other than *K. pneumoniae* and *K. aerogenes*, *Enterobacter* spp including *Klebsiella* (*Enterobacter*) *aerogenes* due to similar resistance mechanism

**e-Table 9**. Microbial concordance of BAL_FAPPP_ vs BAL in patients enrolled with availability of both conventional culture and molecular diagnostics in BAL at VAP suspicion (n° 49). For concordance purpose, the 7 microbiologically confirmed VAP with detection in BAL of isolates not identifiable by BAL_FAPPP_ were considered BAL negative

| BAL_FAPPP_ | BAL | Shared species/ species identified | Patients  N (%) |
| --- | --- | --- | --- |
| **Complete species concordance** |  |  | **13 (26.5)** |
| *Klebsiella pneumoniae* | *Klebsiella pneumoniae* | 1/1 | 2 |
| *Klebsiella aerogenes* | *Klebsiella spp* | 1/1 | 1 |
| *Pseudomonas aeruginosa* | *Pseudomonas aeruginosa* | 1/1 | 3 |
| *Staphylococcus aureus* | *Staphylococcus aureus* | 1/1 | 3 |
| *Haemophilus influenzae* | *Haemophilus influenzae* | 1/1 | 1 |
| *Escherichia coli/Staphylococcus aureus* | *Escherichia coli/Staphylococcus aureus* | 2/2 | 2 |
| *Klebsiella spp/ Pseudomonas aeruginosa* | *Klebsiella spp/ Pseudomonas aeruginosa* | 2/2 | 1 |
| **Partial species concordance** |  |  | **6 (12.2)** |
| *Haemophilus influenzae/ Pseudomonas aerugiona* | *Pseudomonas aeruginosa* | 1/2 | 1 |
| *Proteus spp/Staphylococcus aureus* | *Staphylococcus aureus* | 1/2 | 1 |
| *Streptococcus pneumoniae/Staphylococcus aureus* | *Streptococcus pneumoniae* | 1/2 | 1 |
| *Enterobacter spp/ Staphylococcus aureus* | *Staphylococcus aureus* | 1/2 | 1 |
| *Acinetobacter baumannii complex/ Klebsiella pneumoniae/ Streptococcus agalatiae* | *Acinetobacter baumannii complex/ Klebsiella pneumoniae* | 2/3 | 1 |
| *Proteus spp/ Serratia marcescens/ Staphylococcus aureus* | *Serratia marcescens/ Staphylococcus aureus* | 2/3 | 1 |
| **Total species discordance** |  |  | **10 (20.4)** |
| *Negative* | *Klebsiella spp* | 0/1 | 1 |
| *Staphylococcus aureus* | *Negativo* | 0/1 | 3 |
| *Serratia marcescens* | *Negativo* | 0/1 | 1 |
| *Pseudomonas aeruginosa* | *Negativo* | 0/1 | 3 |
| *Escherichia coli /Staphylococcus aureus* | *Negativo* | 0/2 | 1 |
| *Escherichia coli /Haemophilus influenzae* | *Negativo* | 0/2 | 1 |
| **Both negative samples (VAP suspicion not confirmed)** |  | | **20 (40.9)** |

Legend: BAL_FAPPP_ bronchoalveolar lavage – molecular diagnostics (Filmarray Pneumonia Panel Plus), BAL bronchialveolar lavage - conventional culture, *Klebsiella* spp *Klebsiella* species other than *K. pneumoniae* and *K. aerogenes*, *Enterobacter* spp including *Klebsiella* (*Enterobacter*) *aerogenes* due to similar resistance mechanism

**e-Table 10**. Leukocyte subpopulations in BAL and PB of patients with sVAP, overall and stratified according to microbiological confirmation

| **Leukocyte subpopulations ^a^** | **BAL** | | **PB** | |
| --- | --- | --- | --- | --- |
|  | *VAP (N=24)* | *no-VAP (N=21)* | *VAP (N=27)* | *no-VAP (N=21)* |
| % Lymphocytes | 2.0 (0.7-11.9) | 7.2 (2.4-41.8)* | 4.6 (2.9-8.4) | 5.8 (3.5-9.3) |
| % Monocytes | 5.4 (2.6-7.4) | 9.0 (4.7-14.4)* | 5.4 (4.3-8) | 7 (4.8-9.2) |
| % Neutrophils | 85.2 (73.4-89.8) | 66.7 (33.5-86.4)** | 86.7 (79.7-89.6) | 84.1 (78.7-86) |
| *Lymphocytes, cells/μl* | 16 (8.5-33.5) | 22.0 (11-41) | 550 (317.0-870) | 607 (400-891) |
| % T lymphocytes CD3+ | 93.5 (90-96.8) | 95.4 (93.5-98.2) | 380 (209-637) | 565 (243-722) |
| % T lymphocytes CD4+ | 40.5 (28.9-56.7) | 40 (27.2-45.7) | 222 (130-440) | 342 (148-471) |
| % T lymphocytes CD8+ | 38.6 (28-52.9) | 44.2 (32.9-58.6) | 93 (51-195) | 134 (84-224) |
| % B lymphocytes CD19+ | 0.8 (0.4-1.6) | 0.4 (0.2-1.5) | 105 (60-177) | 70 (28-174) |
| % NK lymphocytes | 3.7 (1.8-6.6) | 2.3 (1.0-3.4) | 40 (15-74) | 36 (29-83) |
| % CD4+/HLA-DR+ lymphocytes | 19.7 (12.5-42.4) | 26 (19.2-41) | 3.4 (2.0-7.2) | 4 (2.1-7.1) |
| % CD8+/HLA-DR+ lymphocytes | 46.2 (23.6-55.8) | 39.7 (18.9-56.8) | 10 (6.4-17.7) | 13.6 (5-36.8) |
| *Monocytes, cells/μl* | 19.5 (10.5-59.5) | 21.5 (9-56.5) | 600 (370-1048) | 854 (528-999) |
| % HLA-DR+ monocytes | 86.1 (73-92.2) | 92.1 (83.7-96.2) | 19.4 (11.7-27) | 25.4 (14.2-39) |
| % classical-monocytes | 32.8 (17.6-50.4) | 32.1 (12.4-45.9) | 87 (83.8-88.5) | 85.3 (83.2-89) |
| % intermediate-monocytes | 47.3 (37.6-73.1) | 64.6 (44.8-81.1) | 11 (8.8-13.1) | 11.6 (7.9-13.9) |
| % non classical-monocytes | 6.1 (3.8-9.4) | 5.6 (2.1-8.8) | 2.4 (1.4-3.6) | 2.3 (1.5-3.1) |
| *Neutrophils, cells/μl* | 474 (127-1297) | 133 (23-1054) | 9449 (6500-12450) | 9465 (6750-11921) |
| % mature neutrophils | 12.8 (7.1-31.1) | 18.5 (11-28.2) | 97.3 (95.2-98.5) | 97.3 (94.9-98.4) |
| % banded neutrophils | 1.7 (0.6-3.2) | 1.2 (0.7-4.1) | 0.7 (0.3-1.8) | 0.8 (0.3-1.3) |
| % progenitors neutrophils | 8.6 (5.6-20.9) | 7.3 (2.3-13.2) | 1.2 (0.6-2.7) | 1.0 (0.5-2.4) |
| % suppressor neutrophils | 64.1 (50.8-77.5) | 61.6 (49.8-79.6) | 0.3 (0.2-0.7) | 0.3 (0.2-0.7) |
| Oxydative burst in neutrophils, MFI |  |  | 26262 (20113-29460) | 27181 (22785-42860) |
| Oxydative burst in monocytes, MFI |  |  | 3249 (2628-4738) | 4081(2925-4827) |

Legend: BAL bronchoalveolar lavage, PB peripheral blood, MFI Mean Fluorescence Intensity.

^a^ Only variables present in at least 43 patients were considered in the table; ^b^ After applying FDR correction, p-values were equal to 0.196; ^c^ After applying FDR correction, p-value was 0.154

*crude p value = 0.03, not significant applying FDR; ** crude p value = 0.01, not significant applying FDR

**e-Table 11**. Cytokines in BAL and peripheral blood of patients with sVAP, overall and stratified according to microbiological confirmation

|  | **BAL**  **N=49** | | **PB**  **N=49** | |
| --- | --- | --- | --- | --- |
| **Cytokines^a^** | **VAP**  **N=27** | **no-VAP**  **N=22** | **VAP**  **N=27** | **no-VAP**  **N=22** |
| CXCL-10, pg/ml | 23.6 (4.5-96.7) | 21.6 (9.0-98.5) | 15.7 (9.0-41.6) | 26.3 (11.5-47.0) |
| IL-6, pg/ml | 51.6 (16.4-129.3) | 33.4 (8.0-95.6) | 24.5 (9.2-49.6) | 31.7 (16.9-84.5) |
| MIP-1 alpha, pg/ml | 39.6 (32.7-69.8) | 36.2 (29.8-59.5) | - | - |
| IL-1 alpha, pg/ml | 2.7 (1.7-11.2) | 2.0 (1.6-11.8) | - | - |
| IL-1 beta, pg/ml* | 135.0 (11.0-449.8) | 10.0 (2.9-105.5) | - | - |
| IL-2, pg/ml | 12.7 (10.8-22.2) | 15.4 (9.5-43.9) | - | - |
| IL-7, pg/ml | - | - | 1.9 (1.4-3.2) | 2.1 (1.4-3.8) |
| TNF-alpha, pg/ml | - | - | 5.2 (2.1-9.5) | 5.7 (3.4-9.4) |

Legend: BAL bronchoalveolar lavage, PB peripheral blood

^a^ Only variables present in at least 43 patients were considered in the table; *crude p value = 0.03, not significant applying FDR
